# Characterisation of the thermal and non-thermal stress conditions that activate the *Plasmodium falciparum* AP2-HS-dependent heat-shock response

**DOI:** 10.1371/journal.ppat.1014346

**Published:** 2026-07-09

**Authors:** Neus Ràfols, Prince B. Nyarko, Marc Chillarón-Adán, Elisabet Tintó-Font, Alfred Cortés

**Affiliations:** 1 Malaria and Neglected Parasitic Diseases Programme, ISGlobal, Barcelona, Catalonia, Spain; 2 Universitat Pompeu Fabra (UPF), Barcelona, Catalonia, Spain; 3 Facultat de Biologia, Universitat de Barcelona (UB), Barcelona, Catalonia, Spain; 4 ICREA, Barcelona, Catalonia, Spain; University of Geneva Faculty of Medicine: Universite de Geneve Faculte de Medecine, SWITZERLAND

## Abstract

To preserve proteome homeostasis and survive at higher-than-optimal temperatures, organisms have evolved the conserved heat-shock (HS) response (HSR), characterised by increased expression of specific chaperone-encoding genes. In the human blood, malaria parasites are frequently exposed to elevated temperatures associated with host fever episodes. The protective HSR of *Plasmodium falciparum*, the parasite that produces the vast majority of malaria clinical cases and deaths, is regulated by the transcription factor AP2-HS. Here, we systematically investigated the conditions that trigger the AP2-HS-dependent HSR and found that even mild HS conditions that do not compromise parasite viability can activate this response. As in other organisms, activation of the HSR in *P. falciparum* is rapid, as it was observed after a HS of only 10 min. Dihydroartemisinin (DHA), a drug that produces general proteome damage, also triggered the HSR, indicating that activation of the malarial HSR is not restricted to thermal stress. The AP2-HS-dependent HSR can be activated in all asexual blood stages, with the exception of very young rings, but not in intermediate or mature gametocyte stages. Accordingly, these gametocyte stages are highly sensitive to HS. Since mature gametocytes are the only stage that can mediate human-to-mosquito transmission, these results suggest that malaria patients with high fever may become transiently non-infectious.

## Author summary

The most common clinical symptom of malaria is periodic fever. To survive at febrile temperatures, malaria parasites must protect themselves from the damage that these temperatures produce, especially on proteins. This is achieved by activating a transcriptional program called the heat-shock response, which consists of increasing the expression of genes encoding molecular chaperones (proteins that assist the folding of other proteins). Here we defined the conditions that trigger the *P. falciparum* protective heat-shock response at different stages of its life cycle. We found that even conditions that mimic a mild fever episode and do not produce substantial damage to the parasite can activate this response, indicating that the parasite uses it in a “preventive” manner. Drugs that produce damage to parasite proteins also activate this response. However, parasites at stages that can transmit the infection from humans to mosquitoes, *i.e.*, gametocytes, cannot activate this response, which reveals a potential parasite vulnerability.

## Introduction

Malaria continues to be one of the most important human infectious diseases, with ~260 million cases and ~600,000 deaths reported every year. *Plasmodium falciparum* causes the most severe form of human malaria and accounts for most clinical malaria cases and deaths [1]. Humans are infected through the bite of an infected female *Anopheles* mosquito. After exponential multiplication in the liver, parasites at the merozoite stage are released and start the intraerythrocytic developmental cycle (IDC). Merozoites invade red blood cells (RBCs) and progress through the ring, trophozoite and schizont stages, the latter characterised by nuclear replication that leads to the formation of up to 32 new merozoites. Approximately 48 h after RBC invasion, schizonts burst, releasing merozoites that invade new RBCs and start the IDC again. At each round of the IDC, a small percentage of the parasites exit this replicative cycle and convert into non-replicative sexual forms called gametocytes, which sequester in the bone marrow for ~10 days, where they develop until they re-enter the peripheral circulation as mature male or female gametocytes [2]. These are the only stages that can infect a mosquito during a bloodmeal, therefore playing an essential role for transmission.

Cyclical fever is the hallmark of clinical malaria. Malaria fever peaks typically last a few hours, with high temperature spikes of up to 41 ºC that usually last ~1 h. In *P. falciparum* infections, fever peaks typically show a periodicity of ~48 h (tertian fever) and coincide with schizont rupture at the end of each round of the IDC [3–5]. The release of malaria ‘toxins’ during schizont bursting, such as hemozoin and glycosylphsophatidylinositol (GPI), triggers the febrile response [6]. Since the duration of the IDC varies between different *Plasmodium* species, the periodicity of fever peaks also varies and is a distinctive trait of each species. However, erratic patterns of malaria fever are also commonly observed, possibly due to asynchronous or multiple infections [4,5,7]. Malarial fever is only triggered above a certain level of parasitaemia, called the pyrogenic threshold [4,5,8]. Therefore, since repeated exposure to malaria results in progressive acquisition of immunity and lower parasite densities, in endemic areas malarial fever (as well as other clinical symptoms) is mainly observed in children [9].

Temperatures above the optimal growth temperature of an organism, including those occurring during fever episodes, can lead to the accumulation of misfolded and aggregated proteins, which can cause cell damage and death. Therefore, all organisms need mechanisms in place to protect themselves from heat-mediated damage. The universal mechanism to prevent or restore proteostasis at higher-than-optimal temperatures, common to both prokaryotes and eukaryotes, is called the heat-shock (HS) response (HSR). It mainly consists of a rapid increase in the expression of chaperone-encoding genes to prevent and revert heat-induced proteome damage [10,11]. In most eukaryotes, from yeast to humans, the HSR is regulated by the conserved transcription factor HSF1 [12–14]. However, HSF1 is absent from malaria parasites, which are regularly exposed to febrile temperatures and therefore must have a HSR.

We recently described a *P. falciparum* transcription factor of the ApiAP2 family, AP2-HS, which activates the malarial HSR [15]. While several parasite transcription factors regulate developmental progression or transitions [16], AP2-HS is the only one known to drive a directed transcriptional response to an environmental condition [5,15]. Although the sequence and structure of AP2-HS is unrelated with HSF1, it plays an analogous role in the regulation of the protective HSR. Three genes are rapidly upregulated in response to febrile temperatures in an AP2-HS-dependent manner: the conserved chaperone-encoding genes *hsp70–1* and *hsp90*, and the gene of unknown function *PF3D7_1421800* [15]. This is an extremely compact HSR compared with the HSF1-dependent HSR described in other eukaryotes, which involves dozens or hundreds of genes [14]. Experiments using recombinant proteins [17] and ChIP-seq [15,18] showed that AP2-HS recognises the tandem G-box DNA motif in the promoters of the *hsp70–1* and *hsp90* genes, which are direct targets of AP2-HS. In contrast, ChIP-seq experiments revealed that AP2-HS does not bind the *PF3D7_1421800* promoter, which lacks a tandem G-box, indicating that this gene is not a direct AP2-HS target [15]. Besides the AP2-HS-dependent HSR, other transcriptional changes occur during HS in an AP2-HS-independent manner [15,19,20], although it is unclear which of these changes are part of a protective response and which ones reflect cell damage or death [5]. HS also increases parasite protein export and cytoadherence [21,22]. Furthermore, several other *P. falciparum* proteins [20,23–27], as well as protein modifications and metabolites [28,29], are needed for HS survival. Some of these factors are ubiquitously present and their levels do not increase during HS; therefore, they cannot be considered part of a response [5].

Parasites lacking AP2-HS show increased sensitivity to HS and also to dihydroartemisinin (DHA), the active metabolite of the frontline antimalarial drug artemisinin and its derivatives (ART) [15]. This result suggests that AP2-HS and the HSR may play a role in ART resistance. Of note, the mode of action of ART involves alkylation of proteins and lipids and formation of reactive oxygen species, which results in general proteome damage, similar to HS, among other consequences for the cell [30,31]. ART-mediated parasite killing requires activation of the drug by haemoglobin degradation products. Mutations in the K13 protein underlie ART resistance by reducing haemoglobin uptake and drug activation in early ring stage parasites [32–35]. However, functional stress responses are also required for ART resistance, as ART still generates cellular stress in K13 mutants [31]. While the importance of the endoplasmic reticulum (ER)-based unfolded protein response (UPR) for ART resistance is well established [36–38], whether the other main cellular stress response, the cytoplasm-based HSR, plays a role in ART resistance remains unknown.

Our previous work revealed that AP2-HS is the regulator of the malarial protective HSR and identified the genes that are activated during HS in an AP2-HS-dependent manner, providing suitable markers to study the HSR. However, the conditions that can or cannot activate the HSR were not investigated, because the HSR was studied only for a fixed, severe HS condition. Characterising which conditions activate the HSR is important to understand the actual physiological relevance of this survival mechanism and to guide the design of future studies investigating the activation of the HSR in human malaria infections. Here we set out to describe the specific conditions that activate the AP2-HS-dependent HSR. We exposed parasites at different stages of their life cycle to different thermal stress conditions, mimicking febrile episodes of different intensity and duration, and assessed the impact on parasite survival and activation of the HSR. We also investigated activation of the AP2-HS-dependent HSR by exposure to drugs.

## Results

### HS duration and temperature conditions needed for activation of the AP2-HS-dependent HSR

**Fig 1:**
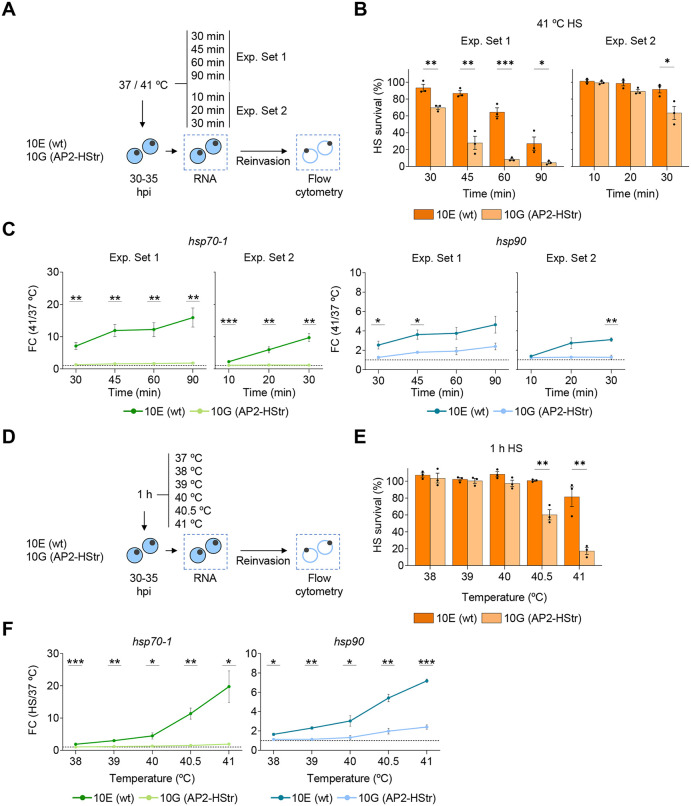
Effect of HS duration and temperature on the activation of the AP2-HS-dependent HSR. **A.** Overview of the experiments to test the effect of HS duration on the activation of the HSR. Tightly-synchronised 10E (wt) and 10G (AP2-HStr) 30-35 hpi (late trophozoite/early schizont stage) cultures were exposed to a 41 ºC HS of variable duration in a water bath. Different durations were tested in two independent sets of experiments, as indicated, with the 30 min time point included in both sets. RNA for transcriptional analysis (by RT-qPCR) was collected immediately after HS. To estimate HS survival, parasitaemia was determined by flow cytometry after reinvasion. Control cultures (no HS) were incubated in parallel in a water bath at 37 ºC for the duration of the HS. **B.** HS survival after exposing cultures to a 41 ºC HS of variable duration, relative to control cultures (no HS). **C.** Fold-change (FC) of *serrs*-normalised *hsp70-1* and *hsp90* transcript levels in cultures exposed to a 41 ºC HS of variable duration relative to transcript levels in control cultures (no HS). The horizontal dotted line indicates a FC of 1 (no change). **D.** Overview of the experiments to test the effect of HS temperature on the activation of the HSR, as in panel A but with HS of a constant duration of 1 h and variable temperature. **E.** HS survival after exposing cultures to a 1 h HS at different temperatures, relative to control cultures (no HS). **F.** Fold-change (FC) of *serrs*-normalised *hsp70-1* and *hsp90* transcript levels in cultures exposed to a 1 h HS at different temperatures, relative to transcript levels in control cultures (no HS). In all panels, values are the mean ± s.e.m. of n = 3 independent biological replicates. Statistically-significant differences between 10E and 10G, calculated using two-sided unpaired Student’s *t*-tests, are indicated by asterisks (*: 0.01 < *P* ≤ 0.05; **: 0.001 < *P* ≤ 0.01; ***: *P* ≤ 0.001).

We developed a new water bath-based standard HS assay to study the effect of febrile temperatures on *P. falciparum in vitro*. The assay and its rationale are described in detail in the Methods section. The standard conditions of the new HS assay are incubation at 41 ºC for 1 h, a temperature and duration similar to the peak of a malaria high-fever episode [3–5]. Using variations of these standard conditions, we investigated how the duration and temperature of a HS affect parasite viability and activation of the HSR. The assays were performed with two 3D7-A subclones: 10E, a subclone with wild type (wt) AP2-HS, and 10G, a subclone with a spontaneous premature STOP codon in AP2-HS that results in a truncated protein lacking the third AP2 domain (AP2-HStr) [15]. The 10G line does not have any growth defect at 37 ºC, but is unable to activate the AP2-HS-dependent HSR and has lower HS survival than 10E [15]; therefore, it provides an ideal control to distinguish AP2-HS-dependent from -independent transcriptional changes after HS. We exposed tightly synchronised (5 h age window) cultures of both subclones at the late trophozoite/early schizont stage [30–35 h post-invasion (hpi)] to a HS of different duration or at different temperature and, at the following cycle, measured parasitaemia by flow cytometry to estimate HS survival. RNA was collected immediately after HS to determine transcript levels of the previously identified AP2-HS target genes *hsp70–1* and *hsp90* by reverse transcription (RT)-quantitative PCR (qPCR), as a proxy for activation of the HSR. For all experiments, control cultures were maintained in parallel in a water bath at 37 ºC during HS (Fig 1A, 1D).

Increasing duration of HS at 41 ºC, from 30 to 90 min, progressively reduced the survival of wt cultures (10E), with essentially 100% survival after a 30 min HS and only ~27% after a 90 min HS. The parasite line expressing truncated AP2-HS (10G) had lower survival than 10E after HS of any duration, with more marked differences for HS of 45 min or longer (Fig 1B, left panel). In 10E, activation of *hsp70–1* and *hsp90*, measured as the transcript levels fold-change (FC) in cultures exposed to HS relative to control cultures, was observed for HS of any duration (30–90 min) and increased with longer HS, whereas in 10G there was essentially no activation (Fig 1C and S1A Fig). The magnitude of *hsp70–1* activation was larger than that of *hsp90* (maximum activation ~16-fold vs ~ 5-fold, respectively), as previously observed [15], although both genes followed a similar pattern. To confirm these results, we performed experiments using the former standard HS assay (incubator-based HS assay), which revealed a similar pattern of reduced survival and increased activation of the HSR with longer HS (S1B–S1C Fig).

The observation of a ~ 7-fold increase in *hsp70–1* transcript levels after a HS of only 30 min prompted us to investigate the effect of a shorter HS in a separate set of experiments. A ≤ 20 min HS did not affect survival of either 10E or 10G (Fig 1B, right panel) but activated the AP2-HS-dependent HSR in 10E, with a ~ 2-fold or ~6-fold increase in *hsp70–1* transcript levels after HS of only 10 or 20 min, respectively (Fig 1C and S1A Fig). Therefore, rapid upregulation of target gene expression, which is a distinctive feature of the HSR in model organisms [10,14], appears to be conserved in *P. falciparum*. This is, to our knowledge, the fastest transcriptional response described to date in malaria parasites.

Next, we assessed the effect of a 1 h HS at different temperatures on parasite survival and activation of the HSR. A 1 h HS at 40 °C or lower temperatures did not affect the viability of either 10E or 10G, whereas at higher temperatures (up to 41 ºC) 10G showed a marked decrease in survival and 10E was only moderately affected (Fig 1D-1E). The magnitude of the HSR was temperature-dependent, with increased *hsp70–1* and *hsp90* transcript levels observed at temperatures of 38 °C or higher in 10E and only very low-level activation in 10G (Fig 1F and S1D Fig). Together with the experiments on HS duration, these results show that HS conditions that do not have an impact on parasite viability even in the 10G line (*e.g.*, < 30 min at 41 ºC or <40.5 ºC for 1 h, Fig 1B, 1E) activate the HSR in parasite lines with fully functional AP2-HS (*i.e.*, 10E).

### Analysis of parasite development and viability after HS

**Fig 2:**
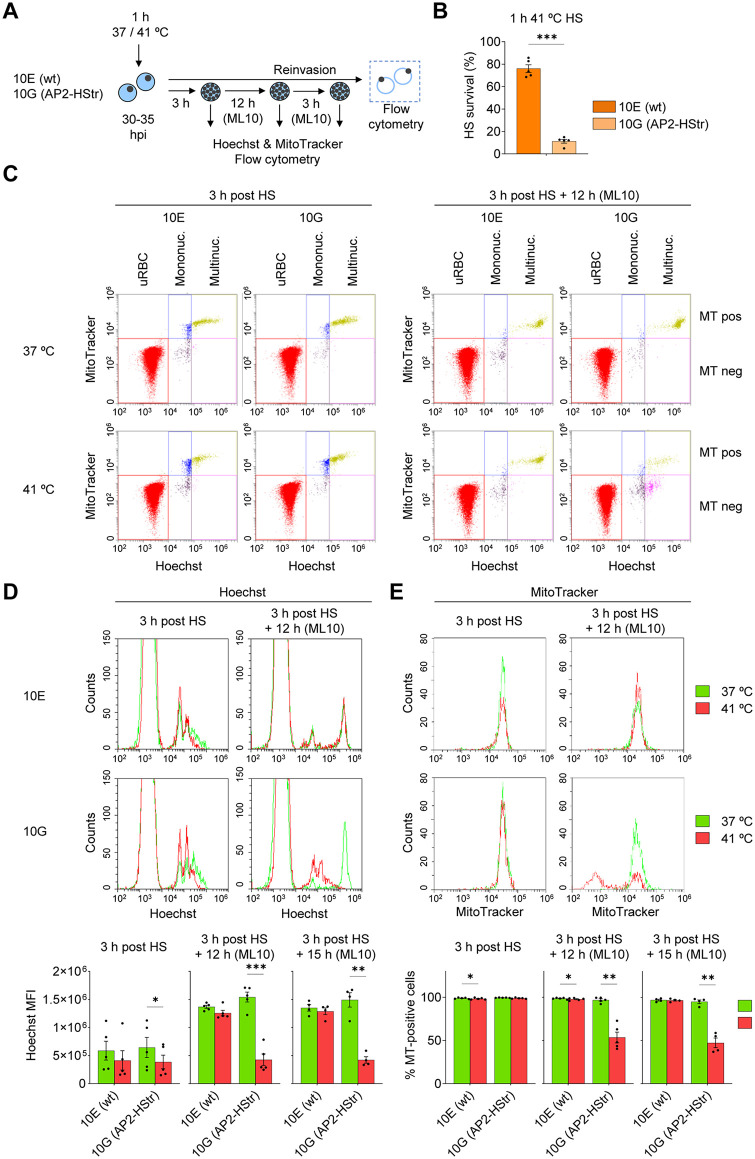
Flow cytometry analysis of parasite DNA content and viability after HS. **A.** Overview of the experiments to test the effect of HS on parasite development and viability. After exposing cultures to HS or control temperature (37 ºC), an aliquot was left unperturbed to assess parasitaemia at the next cycle by flow cytometry (Reinvasion). The rest of the culture was analysed by flow cytometry after 3 h (with Hoechst to measure DNA content and MitoTracker to measure viability) and then cultured with ML10 (inhibits schizont bursting) and analysed at two additional time points. **B.** HS survival (after reinvasion) of cultures exposed to a HS at 41 ºC for 1 h, relative to control cultures (no HS). **C.** Representative Hoechst – MitoTracker (MT) flow cytometry scatter plots of cultures exposed to a HS at 41 ºC for 1 h (or controls at 37 ºC) and then cultured for 3 h (3 h post HS) or for 3 h plus an additional 12 h with ML10 [3 h post HS + 12 h (ML10)]. The gates to define uninfected (uRBCs), mononucleated (infected with rings or trophozoites) or multinucleated (infected with schizonts) RBCs, as well as MitoTracker positive or negative cells (MT pos or MT neg, respectively), are indicated. **D.** Representative histograms of Hoechst signal (top) and quantification of the mean fluorescence intensity (MFI) among multinucleated cells (bottom), at different times after HS. **E.** Representative histograms of MitoTracker signal among multinucleated cells (top) and quantification of the proportion of MitoTracker-positive multinucleated cells (bottom), at different times after HS. In panels C-E, images are representative of n = 5 independent biological replicates. In the bar charts in panels B and D-E, values are the mean ± s.e.m. of the five replicates. Statistically-significant differences between 10E and 10G (panel B), calculated using two-sided unpaired Student’s *t*-tests) and between 37 and 41 ºC (panels D-E), calculated using two-sided paired Student’s *t*-tests), are indicated by asterisks (*: 0.01 < *P* ≤ 0.05; **: 0.001 < *P* ≤ 0.01; ***: *P* ≤ 0.001).

To gain insight into how HS kills parasites expressing wt or truncated AP2-HS, we performed a standard HS assay (30–35 hpi 10E and 10G cultures, HS at 41 ºC for 1 h in a water bath, control cultures incubated in parallel at 37 ºC), and analysed them by flow cytometry at different times after HS. We monitored DNA content using Hoechst and mitochondrial activity using MitoTracker, a marker of viability that only stains live cells [39–41] (Fig 2A-B and S2A Fig). At 3 h after exposure, there was a mixture of multinucleated schizonts and mononucleated parasites, consisting of trophozoites and, in some experiments, also some new rings (Fig 2C, “3 h post HS” graphs). To exclude new rings from the analysis, we calculated the Hoechst mean fluorescence intensity (MFI) only for multinucleated cells. In cultures exposed to HS, the DNA content (Hoechst signal) 3 h post HS was slightly lower than in controls (less than 50% reduction), although the difference was statistically significant only for 10G (Fig 2D, “3 h post HS” graphs). This result indicates a small delay in life cycle progression produced by HS, consistent with previous reports [15]. Mononucleated parasites are not efficiently stained with MitoTracker, possibly because young parasites have low mitochondrial activity, but essentially all multinucleated cells were clearly stained in control cultures (Fig 2C). Therefore, we also performed the MitoTracker analysis including only multinucleated cells. At 3 h after exposure, the vast majority of multinucleated cells were MitoTracker positive under both conditions (41 or 37 ºC) in 10E as well as 10G, indicating that parasites did not directly die during HS or in the following 3 h (Fig 2C, 2E, “3 h post HS” graphs).

To analyse DNA content and viability at the end of the IDC, 3 h after exposing cultures to HS (or to 37 ºC) the ML10 compound was added to inhibit schizont bursting [42,43]. At 12 or 15 h after adding ML10 (46–51 and 49–54 hpi), in 10E the DNA content (Hoechst signal intensity) was similar between cultures exposed to HS and control cultures, indicating that both had successfully completed schizont development and had a similar number of merozoites per schizont (Hoechst signal is a proxy for DNA content and therefore for the number of merozoites per schizont) [Fig 2C-2D, “3 h post HS + 12 h (ML10)” and “3 h post HS + 15 h (ML10)” graphs]. Essentially all 10E schizonts were MitoTracker positive, both in cultures exposed to HS and in control cultures (Fig 2C, 2E), reflecting the low HS sensitivity of 10E. In contrast, at these late time points, 10G cultures exposed to HS showed a major reduction in DNA content compared with controls at 37 ºC, with essentially no increase in DNA content between 3 and 3 + 12 or 3 + 15 h post HS samples (Fig 2C-2D). This observation indicates that 10G cultures were largely unable to replicate DNA and to continue IDC progression after HS. Furthermore, in 10G, half of the multinucleated cells were MitoTracker negative at 3 + 12 or 3 + 15 h post HS (Fig 2C, 2E), indicating that they died between the 3 and 3 + 12 h time points.

Consistent with the results of the flow cytometry analysis, light microscopy examination of Giemsa-stained smears revealed that both 10E and 10G cultures exposed to HS contained apparently healthy late trophozoites and early schizonts at 3 h post HS, similar to control cultures. In contrast, at 3 + 12 h post HS, 10E cultures (HS or control) and control 10G cultures contained healthy fully mature schizonts, whereas 10G cultures exposed to HS contained late trophozoites and early schizonts, similar to the 3 h post HS time point. This confirms that 10G cultures exposed to HS did not develop between these two time points. Furthermore, at the 3 + 12 h post HS time point, some parasites in 10G cultures exposed to HS appeared to be pyknotic (S2B Fig). Together, these results indicate that when the AP2-HS-dependent HSR cannot be activated (10G line), the damage produced by HS leads to growth-arrested schizonts that fail to continue IDC progression, and many parasites die before completing the cycle. Parasite death occurred between 3 and 15 h post HS. None of these defects were observed in parasites capable of activating the AP2-HS-dependent HSR (10E line). The small reduction in next generation parasitaemia in 10E cultures exposed to HS (relative to control cultures) does not appear to be attributable to loss of viability during schizont development and may result from other causes (*e.g.*, reduced RBC invasion or egress) that we could not identify.

### The HSR can be activated at most stages of the IDC

**Fig 3:**
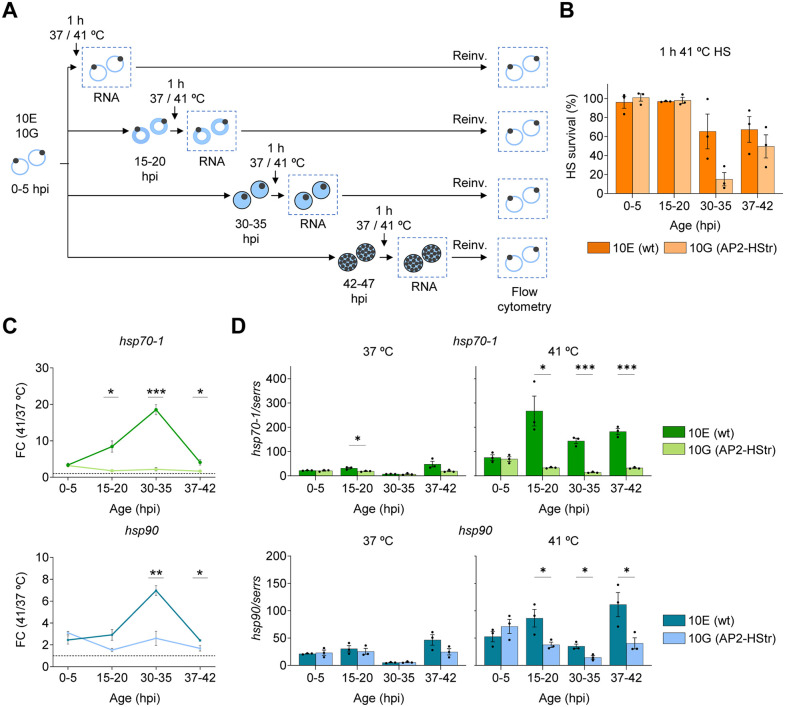
Activation of the AP2-HS-dependent HSR at different stages of the IDC. **A.** Overview of the experiments to test activation of the HSR at different stages of the IDC. Tightly-synchronised 10E (wt) and 10G (AP2-HStr) cultures were exposed to a standard HS at 41 ºC for 1 h in a water bath at different stages, as indicated. RNA for transcriptional analysis (by RT-qPCR) was collected immediately after HS. To estimate HS survival, parasitaemia was determined by flow cytometry after reinvasion (Reinv.). Control cultures (no HS) were maintained in parallel in a water bath at 37 ºC for the duration of the HS. **B.** HS survival after exposing cultures at different stages of the IDC to a HS at 41 ºC for 1 h, relative to control cultures (no HS). **C.** Fold-change (FC) of *serrs*-normalised *hsp70-1* and *hsp90* transcript levels in cultures at different stages of the IDC exposed to a HS at 41 ºC for 1 h, relative to transcript levels in control cultures (no HS). **D.** Transcript levels of *hsp70-1* and *hsp90*, normalised against *serrs* transcripts, in cultures at different stages of the IDC exposed to HS (41 ºC) or not (37 ºC). In all panels, values are the mean ± s.e.m. of n = 3 independent biological replicates. Statistically-significant differences between 10E and 10G, calculated using two-sided unpaired Student’s *t*-tests, are indicated by asterisks (*: 0.01 < *P* ≤ 0.05; **: 0.001 < *P* ≤ 0.01; ***: *P* ≤ 0.001).

To investigate activation of the HSR at different developmental stages, tightly synchronised 10E and 10G cultures were first exposed to a standard HS (1 h at 41 ºC) at four different stages of the IDC (Fig 3A-3B). The next cycle parasitaemia of 10E and 10G cultures exposed at the very early (0–5 hpi) or late (15–20 hpi) ring-stage was minimally affected by HS, consistent with previous reports [15,44,45]. Late trophozoites/early schizonts (30–35 hpi) were the stage most sensitive to HS and showed the largest differences in survival between 10E and 10G, as previously reported [15], whereas schizonts (37–42 hpi) showed survival levels similar to late trophozoites/early schizonts in 10E but were more resistant in 10G.

At most stages, transcript levels of *hsp70–1* and *hsp90* increased after HS in 10E, but not in 10G (Fig 3C). In 10E, late trophozoites/early schizonts showed the highest FC in *hsp70–1* and *hsp90* transcript levels between HS-exposed and control cultures. However, *serine-tRNA ligase* (*serrs*)-normalised *hsp70–1* and *hsp90* transcript levels after HS were similar or even higher in late rings and schizonts than in late trophozoites/early schizonts (Fig 3D). Therefore, the higher FC in late trophozoites/early schizonts is explained by reduced basal expression of *hsp70–1* and *hsp90* at this stage (Fig 3D, 37 ºC panels), as part of the cyclic fluctuations in transcript levels along the IDC characteristic of most *P. falciparum* genes [46]. At the late trophozoite/early schizont stage, to achieve similar transcript levels after HS as at other stages, a more pronounced activation (higher FC) is needed. In very early rings, we detected a ~ 3-fold increase in *hsp70–1* and *hsp90* transcript levels after HS in both 10E and 10G (Fig 3C-3D), which was confirmed (for *hsp70–1*) using *ubiquitin-conjugating enzyme* (*uce*) for normalisation (S3 Fig). These results suggest that, at this stage, *hsp70–1* and *hsp90* may be activated (albeit at low levels) in response to HS independently of AP2-HS. However, we cannot exclude the possibility that expression of the normalising genes may be altered after HS in very early rings, explaining the apparent increase in *hsp70–1* and *hsp90* transcripts. At other stages, differences between 10E and 10G and genome-wide transcriptomic analysis [15] exclude the possibility that changes in *hsp70–1* and *hsp90* are explained by altered expression of the normalising gene.

### Gametocytes at intermediate or late stages of development do not activate the HSR

We also investigated activation of the HSR at different stages of gametocyte development. Given that 3D7-A-derived lines, such as 10E and 10G, have a defect in the *gdv1* gene and do not produce gametocytes [47], for these experiments we used other parasite lines. Since no parasite line was available to produce highly enriched and synchronised populations of gametocytes of all stages, we used two different parasite lines for experiments with early or late gametocyte stages. For both parasite lines, heat-shock survival and activation of the HSR in asexual parasites were similar to the 10E line (S4A Fig).

**Fig 4:**
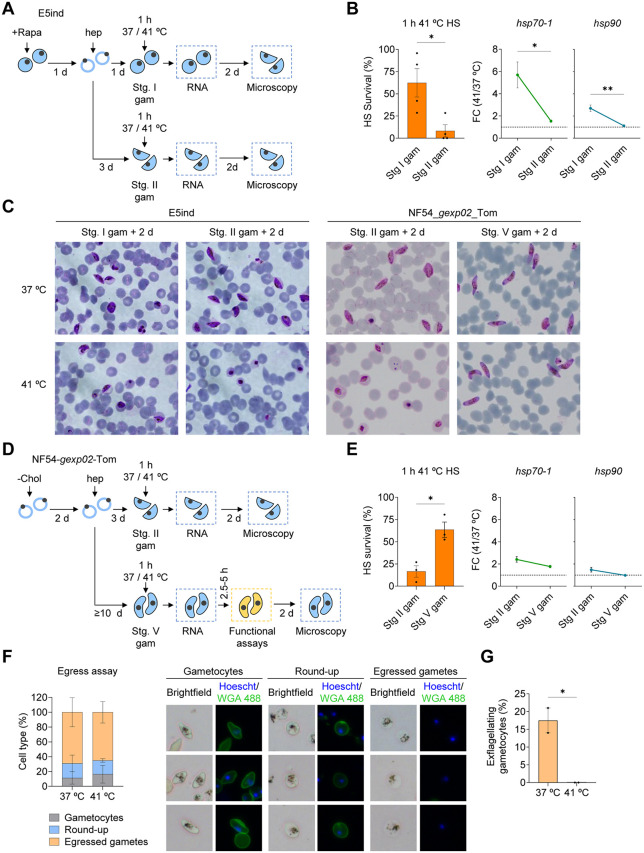
Activation of the AP2-HS-dependent HSR in gametocyes. **A.** Overview of the experiments with the E5ind line to study activation of the HSR at early and intermediate stages of gametocyte development. Sexual conversion was induced by addition of Rapamycin (+Rapa) and, after reinvasion, cultures were maintained with heparin (hep) to prevent asexual growth. Cultures were exposed to a standard HS at 41 ºC for 1 h in a water bath at stage I or stage II of gametocyte development. RNA for transcriptional analysis (by RT-qPCR) was collected immediately after HS. To estimate HS survival, gametocytaemia was determined by light microscopy 2 days after HS. Control cultures (no HS) were maintained in parallel in a water bath at 37 ºC for the duration of the HS. **B.** HS survival and fold-change (FC) of *serrs*-normalised *hsp70-1* and *hsp90* transcript levels in E5ind gametocyte cultures exposed to a HS at 41 ºC for 1 h, relative to control cultures (no HS). Values are the mean ± s.e.m. of n = 4 independent biological replicates. **C.** Representative images of Giemsa-stained smears prepared 2 days after HS at different stages of gametocyte development, for cultures exposed to HS (41 ºC) and their controls (no HS, 37 ºC). **D.** Overview of the experiments with the NF54-*gexp02-*Tom line to study activation of the HSR at intermediate and mature stages of gametocyte development. Sexual conversion was induced by choline depletion (-Chol) and, after reinvasion, cultures were maintained with heparin (hep) to prevent asexual growth. Cultures were exposed to a standard HS at 41 ºC for 1 h in a water bath at stage II or stage V of gametocyte development. RNA for transcriptional analysis (by RT-qPCR) was collected immediately after HS. To estimate HS survival, gametocytaemia was determined by light microscopy 2 days after HS. Functional assays with mature gametocytes were performed 2.5-5 h after HS. Control cultures (no HS) were maintained in parallel in a water bath at 37 ºC for the duration of the HS. **E.** HS survival and fold-change (FC) of *serrs*-normalised *hsp70-1* and *hsp90* transcript levels in NF54-*gexp02-*Tom gametocyte cultures exposed to a HS at 41 ºC for 1 h, relative to control cultures (no HS). Values are the mean ± s.e.m. of n = 3 independent biological replicates. However, for stage V gametocyte survival assays, each of the three values is the mean of two HS survival assays performed on aliquots of the same cultures on separate days. **F.** Quantification of gamete activation (round-up) and egress in NF54-*gexp02-*Tom mature (stage V) gametocyte cultures exposed to a HS at 41 ºC for 1 h and control cultures (37 ºC, no HS). The brightfield and fluorescence (blue: Hoechst; green: WGA-Oregon Green 488) images at the right are representative of parasites that were scored as gametocytes (elongated, peripheral WGA signal), round-up (round shape, peripheral WGA signal) or egressed gametes (round shape, no peripheral WGA signal). Values are the mean ± s.e.m. of n = 2 independent biological replicates, with ≥200 cells scored for each sample. **G.** Quantification of the percentage of exflagellating gametocytes in NF54-*gexp02-*Tom mature (stage V) gametocyte cultures exposed to a HS at 41 ºC for 1 h and control cultures (37 ºC, no HS). Values are the mean ± s.e.m of n = 2 independent biological replicates, with 2 technical replicates for each biological replicate. In all panels, statistically-significant differences between different gametocyte stages (panels B, E) or between HS and no HS (panels F, **G)**, calculated using two-sided unpaired Student’s *t*-tests (except for panel F, two-way ANOVA), are indicated by asterisks (*: 0.01 < *P* ≤ 0.05; **: 0.001 < *P* ≤ 0.01; ***: *P* ≤ 0.001).

To study the HSR in early and intermediate gametocytes, we used the 3D7-derived E5 gametocyte-inducible transgenic line (E5ind), in which synchronous sexual conversion of the majority of parasites (typically ~90%) can be induced by addition of rapamycin [47]. HS experiments were performed two and four days after induction with rapamycin, when the vast majority of parasites were at stage I or II of gametocyte development, respectively (Fig 4A). The stage composition of the cultures was confirmed by light microscopy analysis (S4B Fig). Cultures were maintained with heparin to eliminate the few parasites that did not convert into gametocytes. While stage I gametocytes cannot be clearly distinguished from asexual trophozoites by morphology, we previously demonstrated that rapamycin-induced recombination at the *pfap2-g* locus in the E5ind line results in activation of the gene and sexual conversion in ~90% of parasites, whereas the majority of the remaining parasites die [47]. Here we confirmed efficient rapamycin-induced recombination in essentially all parasites using PCR analysis of genomic DNA (S4C Fig). Furthermore, in cultures maintained without heparin, 8 and 21% of the parasites were asexual at days 5 or 7 post-induction, respectively (S4D Fig). Considering that sexual parasites are non-replicative and asexuals had undergone two or three rounds of exponential multiplication during this time, these data suggest that the vast majority of parasites were gametocytes at the time of HS.

E5ind cultures at stage I or II of gametocyte development were exposed to a standard HS (1 h at 41 ºC) and RNA for transcriptional analysis collected immediately after HS (Fig 4A). Control cultures not exposed to HS were always analysed in parallel. Since gametocytes are non-replicative, HS survival was estimated as the gametocytaemia of cultures exposed to HS relative to control cultures, determined by light microscopy analysis of Giemsa-stained smears prepared 2 days after HS. Although these assays showed substantial variability, stage II gametocytes, with a mean survival of ~8%, were clearly more sensitive to HS than stage I gametocytes or any asexual stage (in parasite lines expressing wt AP2-HS) (Fig 4B-4C and S4E Fig). Furthermore, stage II gametocytes showed minimal activation of *hsp70–1* and *hsp90* after HS, whereas stage I gametocytes activated it at rates comparable to asexual stages (Fig 4B and S4F Fig). These results suggest a possible causal relationship between inability to activate the HSR and low survival to HS in stage II gametocytes.

Since the E5ind line does not produce infective male gametocytes [47] and E5ind gametocyte cultures tend to loose synchronicity after stage III, to study the HSR in mature gametocytes we used the NF54-*gexp02*-Tom reporter line [48]. This parasite line was regularly maintained with a choline supplement to repress sexual conversion. After removing choline to allow sexual conversion of a large proportion of the parasites [40,49] and treating cultures with heparin to eliminate asexual parasites, NF54-*gexp02*-Tom stage II or stage V gametocytes were exposed to a standard HS (1 h at 41 ºC) (Fig 4D). Stage II gametocytes showed low HS survival (~17%), similar to E5ind gametocytes at this stage. In contrast, using the same microscopy-based assay, stage V (mature) gametocytes showed higher HS survival (~64%) (Fig 4C-4E and S4E Fig). However, transcriptomic analysis using RNA collected immediately after HS showed minimal activation of the HSR in both stage II and stage V gametocytes (Fig 4E and S4F Fig). Together, these results show that, after stage I, gametocytes have very limited ability to activate the HSR.

Stage V gametocytes, which are a quiescent stage, appeared to be resistant to HS, despite minimal activation of the HSR. To test if their viability was compromised, in spite of normal morphology, we performed functional assays 2.5 to 5 h after HS (Fig 4D and S4G Fig). An activation and egress assay showed that stage V gametocytes exposed to HS rounded-up and egressed from their host RBCs at a similar rate as control (no HS) gametocytes (Fig 4F). However, exflagellation assays revealed total absence of exflagellation centres in HS-exposed cultures, in contrast to the ~ 17% exflagellation rate observed in control cultures (Fig 4G). This result indicates that mature male gametocytes are no longer functional after HS.

### The gene *PF3D7_1421800* is dispensable for HS survival

**Fig 5:**
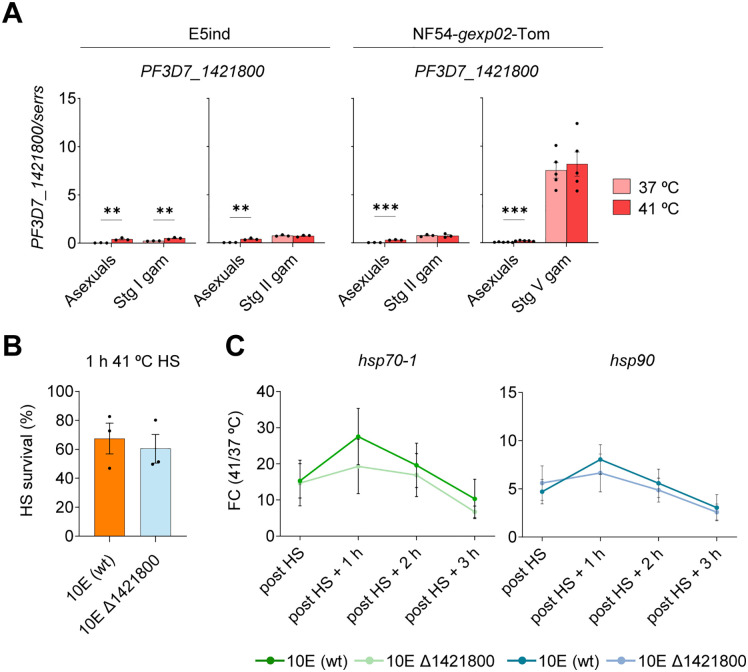
Characterisation of the role of the *PF3D7_1421800* gene in the HSR. **A.** Transcript levels of *PF3D7_1421800*, normalised against *serrs*, in asexual blood stages at the late trophozoite/early schizont stage and different stages of gametocyte development, exposed to a standard HS for 1 h (41 ºC) or not (37 ºC). Values are the mean ± s.e.m. of n = 3-5 independent biological replicates (see individual data points). **B.** HS survival after exposing 30-35 hpi 10E (wt) and 10E_Δ1421800 cultures to a standard HS at 41 ºC for 1 h, relative to control cultures (no HS). Values are the mean ± s.e.m. of n = 3 independent biological replicates. **C.** Fold-change (FC) of *serrs*-normalised *hsp70-1* and *hsp90* transcript levels in 10E (wt) and 10E_Δ1421800 cultures immediately after HS (post HS) or after additional incubation at 37 ºC following HS, relative to transcript levels in control cultures (no HS). Values are the mean ± s.e.m. of n = 3 independent biological replicates. In all panels, statistically-significant differences between HS and no-HS cultures (panel A) or between 10E and 10E_Δ1421800 (panels B-C), calculated using two-sided unpaired Student’s *t*-tests, are indicated by asterisks (*: 0.01 < *P* ≤ 0.05; **: 0.001 < *P* ≤ 0.01; ***: *P* ≤ 0.001).

The *PF3D7_1421800* gene, of unknown function, is activated in an AP2-HS-dependent manner in response to HS [15]. Analysis of *PF3D7_1421800* expression in the samples described in Figs 1 and 3 confirmed that transcript levels for this gene can increase up to 30 times in 10E cultures exposed to HS (relative to control cultures at 37 ºC), but in some experiments a relatively large increase (>10-fold) was also observed in 10G (S5 Fig). Activation in response to HS was only observed in trophozoites and schizonts. Together, these data show that the activation pattern for *PF3D7_1421800* at different stages, durations and temperatures is clearly distinct from *hsp70–1* and *hsp90*. Of note, the basal normalised transcript levels of this gene are very low at all IDC stages (*e.g.*, ~ 300-fold lower than *hsp70–1* at 30–35 hpi). Inspection of expression profiles of this gene in published datasets available in PlasmoDB revealed that the gene is expressed at much higher levels in mature gametocytes than during the IDC, which was confirmed by the analysis of its transcript levels in our gametocyte samples (Fig 5A). However, the expression of the gene was not increased in response to HS in stage II or V gametocytes.

To further investigate the role of this gene in the HSR, we generated a *PF3D7_1421800* knockout (KO) line (10E_Δ1421800) by transfection of 10E cultures (S6A Fig). The KO line had a multiplication rate, HS survival and activation of *hsp70–1* and *hsp90* expression in response to HS similar to the wt parental line (Fig 5B–5C and S6B, S6C Fig). These results indicate that this gene is dispensable for normal progression through the IDC and for the activation of the AP2-HS-dependent protective HSR.

Finally, we explored a possible role of this gene in switching off the HSR. It is well established that activation of the HSR is transient, in *P. falciparum* [15] as well as in other organisms [10,14], possibly because prolonged activation is detrimental for the cells [50]. We speculated that *PF3D7_1421800* may be involved in the downregulation of the HSR, which may explain its activation after HS only in parasites able to activate the HSR, in spite of not being an AP2-HS direct target. However, the *hsp70–1* and *hsp90* transcript levels dynamics at 1–3 h after HS showed no differences between wt and 10E_Δ1421800 cultures, excluding a role for this gene in the deactivation of the HSR (Fig 5C and S6C Fig).

Together, these results suggest that *PF3D7_1421800* is a gametocyte-specific gene that only has residual expression during the IDC. Its intriguing activation in response to HS in late asexual stages is of unclear significance, and not fully dependent on functional AP2-HS.

### A sublethal dose of DHA triggers the AP2-HS-dependent HSR

In a previous study, we showed that deletion of the entire *ap2-hs* gene (Δ*ap2-hs* lines) results in reduced growth at 37 ºC, inability to activate the HSR and even lower HS survival than in parasites with truncated AP2-HS lacking the third AP2 domain [15]. Furthermore, Δ*ap2-hs* lines of different genetic backgrounds showed higher susceptibility to DHA than their wt controls, both at the ring and at the trophozoite stages. This result may suggest that the HSR, which is not activated in Δ*ap2-hs* lines, contributes to DHA survival. Deletion of *ap2-hs* also resulted in increased sensitivity to the proteasome inhibitor epoxomicin. However, the 10G parasite line, which expresses truncated AP2-HS lacking only the third AP2 domain (AP2-HStr) and is also unable to activate the HSR, showed only a very minor increase in sensitivity to DHA at the trophozoite stage and no changes in sensitivity to epoxomicin [15]. These observations raise a possible alternative explanation: Δ*ap2-hs* lines may suffer from constitutive proteome damage, even in the absence of stress, as a consequence of their low basal expression of *hsp70–1* and *hsp90* (which was not observed in 10G) [15]. This could make them more sensitive to additional proteotoxic damage (*e.g.*, by DHA or epoxomicin).

**Fig 6:**
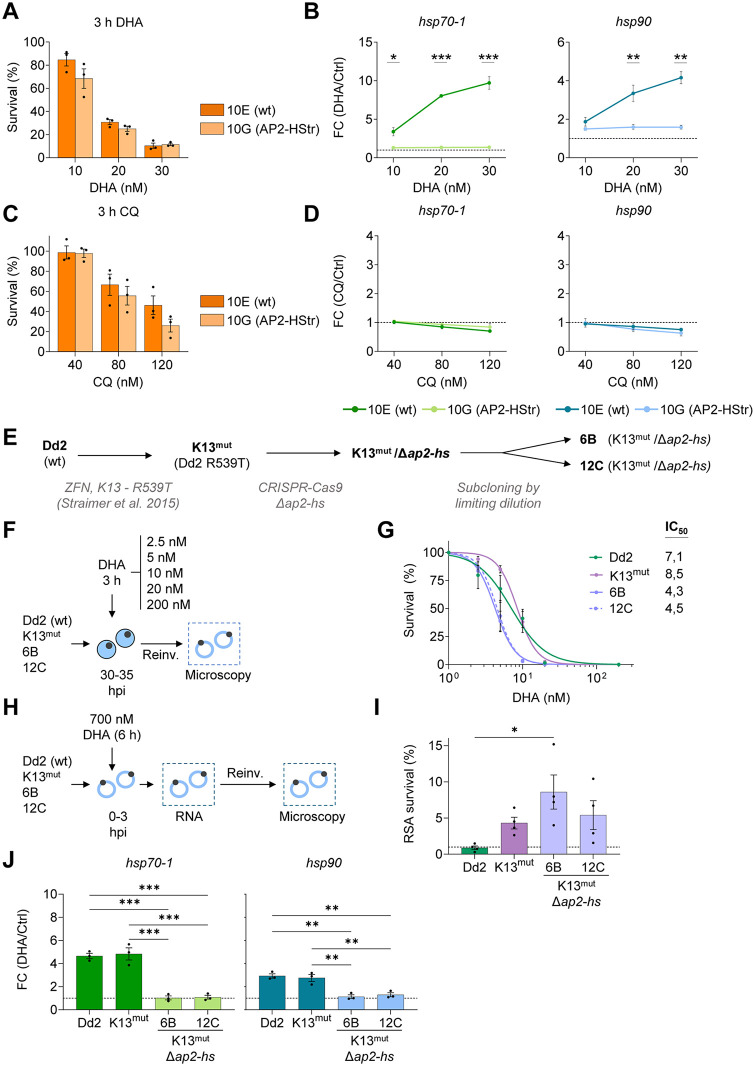
DHA triggers the AP2-HS-dependent HSR. **A.** Survival of 10E (wt) and 10G (AP2-HStr) 30-35 hpi cultures after exposure to a 3 h DHA pulse at different concentrations, relative to controls not exposed to DHA. **B.** Fold-change (FC) of *serrs*-normalised *hsp70-1* and *hsp90* transcript levels in cultures exposed to DHA at different concentrations relative to transcript levels in control cultures (no DHA). In panels A and B, values are the mean ± s.e.m. of n = 3 independent biological replicates. Statistically-significant differences between 10E and 10G, calculated using two-sided unpaired Student’s *t*-tests, are indicated by asterisks (*: 0.01 < *P* ≤ 0.05; **: 0.001 < *P* ≤ 0.01; ***: *P* ≤ 0.001). **C-D.** Same as panels A and B, for experiments with CQ instead of DHA. **E.** Schematic of the parasite lines used for the experiments in the following panels. **F.** Overview of the experiments to determine the IC_50_ of K13 and AP2-HS mutants to DHA. Tightly-synchronised 30-35 hpi cultures were exposed to a 3 h DHA pulse at different concentrations. To estimate survival, parasitaemia was determined by light microscopy after reinvasion (Reinv.). **G.** Survival of 30-35 hpi cultures after exposure to a 3 h DHA pulse at different concentrations, relative to controls not exposed to DHA. Values are the mean ± s.e.m. of n = 2 independent biological replicates. IC_50_ values for each parasite line were calculated using dose-response sigmoidal curves. **H.** Overview of the ring survival assay (RSA) experiments with K13 and AP2-HS mutants. For the RSA, tightly-synchronised 0-3 hpi cultures were exposed to a 6 h 700 nM DHA pulse. RNA for transcriptional analysis (by RT-qPCR) was collected immediately after the pulse. To estimate survival, parasitaemia was determined by light microscopy after reinvasion. **I.** Survival of the different parasite lines in the RSA. Survival above 1% (dotted horizontal line) is considered an ART-resistant phenotype. Values are the mean ± s.e.m. of n = 4 independent biological replicates. **J.** Fold-change (FC) of *serrs*-normalised *hsp70-1* and *hsp90* transcript levels in cultures exposed to DHA relative to control cultures (no DHA) in the RSA. Values are the mean ± s.e.m. of n = 3 independent biological replicates. In panels I and J, statistically-significant differences between parasite lines, calculated using one-way ANOVA, are indicated by asterisks, as in panels A-D.

To directly investigate if DHA activates the HSR, we measured *hsp70–1, hsp90* and *PF3D7_1421800* transcript levels in 30–35 hpi 10E and 10G cultures treated with sublethal doses of DHA (3 h pulse) and untreated controls. Survival was similar between 10E and 10G at all the DHA concentrations tested (Fig 6A). However, dose-dependent activation of *hsp70–1* and *hsp90* expression in response to DHA was evident in 10E (up to 10-fold increase) but not in 10G, whereas *PF3D7_1421800* was only modestly activated in both parasite lines (Fig 6B and S7A–S7B Fig). These results provide evidence for activation of the AP2-HS-dependent HSR by a proteotoxic condition different from thermal stress, despite activation not resulting in substantially increased survival in this case.

To investigate if other drugs can activate the HSR or activation is restricted to stress conditions that induce proteome damage, we exposed 10E and 10G cultures to chloroquine (CQ), a drug that kills parasites with a mode of action that does not primarily involve general proteotoxic stress [30]. The two parasite lines showed similar survival to the different doses of CQ tested (Fig 6C), and none of the doses resulted in an increase in *hsp70–1* or *hsp90* transcript levels in either 10E or 10G (Fig 6D and S7A, S7C Fig). Therefore, activation of the AP2-HS-dependent HSR appears to occur only in response to proteome damage.

### Activation of the AP2-HS-dependent HSR after a high-dose DHA pulse

Having established that a sublethal DHA pulse triggers the AP2-HS-dependent HSR, we set out to investigate if a clinically-relevant dose of DHA also triggers this response. Since all wt parasites exposed to such a dose of DHA die, we performed these experiments using ART-resistant K13 mutants [32–35]. Additionally, with these experiments we aimed to determine if the AP2-HS-driven HSR is needed for ART resistance in K13 mutants.

We generated the double transgenic line K13^mut^/Δ*ap2-hs*, in which the entire *ap2-hs* gene was deleted in the previously described Dd2 R539T (hereafter K13^mut^) line [34]. This parasite line, of Dd2 genetic background, was engineered to introduce an ART-resistance mutation in the K13 protein. Subclones 6B and 12C of K13^mut^/Δ*ap2-hs* were used for all the experiments (Fig 6E and S8A Fig). For consistency with previous studies, K13^mut^/Δ*ap2-hs* subclones and their controls (wt Dd2 and K13^mut^) were regularly maintained at 35 ºC. As observed for other Δ*ap2-hs* lines [15], the K13^mut^/Δ*ap2-hs* subclones showed a reduced multiplication rate (compared to their controls) at either 35 ºC or 37 ºC, were highly sensitive to HS and failed to activate the expression of *hsp70–1*, *hsp90* and *PF3D7_1421800* in response to HS (S8B–S8D Fig). The K13^mut^ line and the parental wt Dd2 line had a similar IC_50_ for DHA at 30–35 hpi, consistent with previous reports showing that ART resistance does not result in changes in the IC_50_ for this drug at the trophozoite stage [51]. In contrast, K13^mut^/Δ*ap2-hs* subclones had a lower IC_50_ (Fig 6F-6G), consistent with previous reports for Δ*ap2-hs* lines [15].

Next, we analysed K13^mut^/Δ*ap2-hs* and control lines using the ring-survival assay (RSA), the gold standard *in vitro* assay to predict ART resistance *in vivo* [51]. In this assay, tightly synchronised cultures at the very early ring stage (0–3 hpi) are exposed to a 6 h DHA pulse at a clinically-relevant concentration (700 nM) and parasitaemia is measured by light microscopy 66 h later to determine the proportion of surviving parasites. While the Dd2 wt line showed a sensitive phenotype (<1% survival), the K13^mut^ and the K13^mut^/Δ*ap2-hs* lines (6B and 12C subclones) showed a resistant phenotype (>1% survival), indicating that the AP2-HS-dependent HSR is not essential for ART resistance in K13 mutants (Fig 6H-6I). The analysis of RNA samples collected immediately after the 6 h DHA pulse showed a clear increase in *hsp70–1* and *hsp90* transcripts in the wt Dd2 and the K13^mut^ lines, but not in the K13^mut^/Δ*ap2-hs* subclones (Fig 6J and S9 Fig). This result demonstrates that a DHA pulse mimicking a clinical ART treatment triggers the AP2-HS-dependent HSR, but this response is not needed for survival of K13 mutants in the clinically-relevant RSA.

## Discussion

We previously characterised the susceptibility of different IDC stages to HS, but activation of the AP2-HS-dependent HSR was only investigated for a fixed severe HS at the late trophozoite/early schizont stage [15]. The minimal thermal or non-thermal conditions needed to activate the HSR were not explored, and no experiments were performed with gametocytes. Here we systematically investigated which conditions activate the *P. falciparum* AP2-HS-dependent HSR, including thermal stress of different duration and intensity and chemical stress induced by the antimalarial drugs DHA and CQ. We also investigated which asexual and sexual parasite blood stages can activate the HSR. We found that both severe and mild thermal stress conditions, including conditions that have essentially no impact on parasite viability, can activate the HSR. Treatment of asexual parasites with DHA also activated the HSR, indicating that the parasite may use the AP2-HS-dependent response to withstand non-thermal types of proteome-damaging stress. The HSR was activated at most stages of the IDC, but not in gametocytes beyond stage I.

We developed a new standard HS assay in which HS is performed in a water bath instead of an incubator. This has important advantages, including improved reproducibility, better concordance between the temperature that is set and the temperature of the cultures and suitability to study HS of short duration (because cultures achieve the target temperature faster). The impact on parasite viability of our new standard HS, 41 ºC in a water bath for 1 h, is similar to the impact of a HS at 41.5 ºC for 3 h in an incubator, our previous standard assay [15]. No single HS conditions can be representative of all malaria fever episodes because malarial fevers exhibit a broad range of duration and temperature, but 1 h at 41 ºC mimics a typical spike during a natural malaria high-fever episode [3–5]. In any case, we extensively tested alternative conditions, which confirmed that the AP2-HS-dependent HSR is activated under a broad range of conditions. These experiments also confirmed that our standard conditions are ideally suited for the molecular characterisation of the HSR, because they result in high-level activation of the HSR and clearly reveal phenotypic differences between wt parasites and AP2-HS mutants. Other studies performed HS in an incubator and used conditions that may be representative of different malaria fever episodes, such as an 8 h HS at 41 ºC over three consecutive cycles (starting with cultures at the ring stage) [20], a 2 h [19] or 6 h [26] HS at 41 ºC on non-synchronised cultures, or a 6 h HS at 40 ºC at the ring stage [52]. The different HS conditions used by different studies, with different impact on parasite survival, complicate the comparison of the transcriptomic changes observed after HS. While there was a strong overlap in the genes with altered transcription after HS between some of the studies, *e.g.,* the study by Oakley et al. [19] and our previous study [15], other studies showed rather divergent results. Furthermore, HS produces many transcriptomic alterations as a consequence of developmental delay, cell damage or cell death, which can mask the identification of the transcriptional changes that are actually part of a protective response [5].

After HS, our standard assay used transcript levels of AP2-HS target genes as a proxy for HSR activation. Transcript levels were analysed immediately after HS because the HSR is transient, in *P. falciparum* [15] as well as in other organisms [10,14]. To determine the impact of HS on parasite viability, our standard assay measures parasitaemia at the next cycle. Flow cytometry experiments with the MitoTracker marker of viability revealed that in parasites unable to mount the AP2-HS-dependent response, the damage produced by HS did not kill parasites immediately, but arrested schizont development and many schizonts died several hours later. Given this complex scenario, we consider that assessing survival at the following cycle provides advantages, as it captures the global effect of HS on parasite viability at different developmental steps. Lastly, to distinguish the AP2-HS-dependent HSR from other transcriptional changes during HS, our experiments included a control parasite line (10G) in which the AP2-HS-dependent HSR cannot be activated. Together, our new assay enabled the sensitive detection of HSR activation with high confidence, even when the magnitude of the response was low.

Using the new HS assay, we found that a HS of only 10 min can lead to low magnitude but consistent activation of the HSR, demonstrating that the malarial HSR is a rapid response, similar to all other eukaryotes in which it has been investigated [10,14]. The rapidity of the response suggests that its activation is mediated by direct changes at the level of the AP2-HS protein, rather than increased expression of *ap2-hs*, similar to HSR activation in other organisms, which involves changes at the level of the HSF1 protein [12–14]. This model is consistent with the results of transcriptomic analyses after HS, which showed no major changes in *ap2-hs* transcript levels [15]. Furthermore, HS at a temperature as low as 38 ºC also led to low level but consistent HSR activation. The conditions that activated the HSR fall well within the range of thermal conditions encountered by the parasite during fever episodes [3–5], suggesting that the parasite frequently activates the HSR during clinical malaria infections. Of note, axillary temperature, which is regularly used in epidemiological studies to measure fever, typically gives values up to 1 ºC below the internal body temperature to which the parasite is actually exposed [53–55], implying that essentially all malaria fevers involve conditions likely to activate the HSR. The observation that activation of the HSR occurs under HS duration and temperature conditions that do not impact parasite viability (even parasites unable to activate the AP2-HS-dependent HSR survive under these conditions) suggests that *P. falciparum* responds to thermal stress in a ‘preventive’ manner. Activating the HSR early during a fever episode, before it actually produces irreversible damage and compromises viability, can provide a survival advantage to the parasite: preventing the formation of misfolded or aggregated proteins, rather than repairing them after they accumulate, is a more effective strategy to survive thermal stress.

Our results confirm previous reports indicating that ring stages are intrinsically resistant to febrile temperatures [15,44,45], even in parasite lines unable to activate the AP2-HS-dependent HSR. In such parasite lines, we found that the level of survival to HS along the IDC somehow parallels the basal levels of *hsp70–1* and *hsp90*: both HS survival and basal expression of these genes are lowest at the late trophozoite/early schizont stage [46,56,57]. This observation suggests that the basal expression levels of these key chaperone-encoding genes may at least in part determine the variability in HS susceptibility between IDC stages. Despite the changes in basal transcript levels of chaperone-encoding genes along the IDC, we demonstrate that the AP2-HS-dependent HSR can be activated at all stages of the IDC except for very early rings. Of note, the HSR can be activated in 15–20 hpi ring stages, in which the response appears to be unnecessary for survival, which again supports the idea of ‘preventive’ activation of the HSR under non-lethal conditions. However, it is possible that the viability of ring stages would be affected by a more severe HS, as previously reported [19,45], and therefore the capacity to activate the HSR response in rings may provide a survival advantage.

In contrast to the capacity of most IDC stages to activate the AP2-HS-dependent HSR, experiments with sexual stages revealed that only stage I gametocytes can activate it, whereas more mature gametocytes cannot. This was paralleled by low HS survival of gametocytes at intermediate or mature stages, revealed either by microscopy analysis of parasites 2 days after HS or by functional assays. These unexpected observations raise the intriguing possibility that, in natural malaria infections, intermediate and mature gametocytes may be killed by high-fever episodes. In the case of intermediate gametocytes (*e.g.*, stage II), which develop in the bone marrow [2], we cannot exclude the possibility that this niche confers some level of physical protection against temperature changes. However, mature (stage V) gametocytes are found in the peripheral circulation and therefore are fully exposed to febrile temperatures. Since stage V male gametocytes exposed to HS are unable to exflagellate and both functional males and females are needed for successful transmission, we predict that malaria patients suffering from high-fever episodes may become transiently non-infectious to mosquitoes. Some published field-based studies also support the idea that febrile patients have reduced infectivity, for both *P. falciparum* [58–60] and *P. vivax* [61] malaria. In contrast, the future infectivity of patients with fever may increase, because febrile temperatures enhance sexual conversion rates [40] and this could result in higher number of infectious gametocytes after maturation (~10 days). These results are suggestive of a complex, double-edged effect of fever on transmission: on the one hand parasites enhance their investment in transmission under stress conditions, as part of a survival strategy, but on the other hand gametocytes are sensitive to temperature. Future studies specifically designed to address the effect of fever on transmission potential should use mosquito infection experiments to confirm whether high fever results in transient non-infectiousness, and establish if there is a temperature threshold above which malaria patients become transiently non-infectious.

We demonstrate that the AP2-HS-dependent HSR is activated in response to DHA treatment, which produces global proteome damage, but not by CQ, which kills parasites by a different mechanism [30]. However, in contrast to the ER-based UPR, which is needed for ART resistance [30,31,36–38], activation of the AP2-HS-dependent HSR does not appear to be essential for ART resistance: first, DHA sensitivity was similar between wt parasites and parasites expressing truncated AP2-HS, which cannot activate the HSR. Second, survival of K13 mutants in the clinically-relevant RSA [51] is similar between *ap2-hs* wt and KO lines. Therefore, the AP2-HS-dependent HSR is activated by both thermal stress and DHA-induced proteotoxic stress, but only activation in response to thermal stress is associated with increased survival. A possible explanation for this observation is that DHA exerts pleiotropic effects on the parasite, such that the drug not only affects proteome integrity but also disrupts lipids, generates reactive oxygen species and interferes with multiple cellular processes through protein alkylation [30,31]. While the upregulation of *hsp70–1* and *hsp90* in response to DHA likely contributes to counteracting proteome damage, it may not protect from the additional damage on other cell components caused by DHA. Thus, the reduced survival of Δ*ap2-hs* lines to DHA sublethal pulses [15] most likely reflects basal proteome damage, which leaves parasites at the edge of proteostasis collapse, rather than inability to activate the HSR.

Overall, we present a detailed characterisation of the conditions that activate the evolutionarily conserved HSR, a major survival mechanism operating at the transcriptional level, in *P. falciparum*. This paves the way to characterise the activation of the response and its physiological relevance in natural malaria infections.

## Materials & methods

### Parasite cultures

The 3D7-A subclones 10E and 10G [62], the gametocyte-inducible line E5ind [47], the NF54-*gexp02*-Tom reporter line [48], and the Dd2 and K13^mut^ (Dd2 R539T) lines [34] have been previously described. Parasites were cultured under standard conditions with either B^+^ or O^+^ RBCs at 3% haematocrit in a 5% CO_2,_ 2% O_2_, balance N_2_ atmosphere. The 10E, 10G, E5ind and 10E_Δ1421800 lines were regularly cultured at 37 ºC under static conditions in standard RPMI-based medium supplemented with Albumax II (Invitrogen). The K13^mut^/Δ*ap2-hs* line (6B and 12C subclones) and its controls (Dd2 and K13^mut^ lines) were maintained under the same conditions but at 35ºC. The NF54-*gexp02*-Tom line was regularly cultured at 37 ºC under shaking conditions with the same culture medium supplemented with 2 mM choline chloride (Sigma-Aldrich C7527) to repress sexual conversion.

### Generation of transgenic parasite lines

To disrupt the *PF3D7_1421800* gene (10E_Δ1421800 line), we used the CRISPR-Cas9 system (S6A Fig). Cloning was performed using the InFusion system (Takara, n. 639642). The donor plasmid (pL6 _HRs1421800_hdhfr) was generated from a modified pL6-egfp-yfcu plasmid [33] in which the *yfcu* cassette was previously removed [47]. The homology regions (HRs) were PCR-amplified from genomic DNA (HR1: positions -460–0 from the start codon of *PF3D7_1421800*; HR2, positions +1 to +439 from the stop codon) and cloned into SpeI/AflII and EcoRI/AatII sites, respectively, replacing the GFP5’ region (HR1) or the GFP3’ region and sgRNA cassette (HR2) in the original plasmid. The Cas9 plasmid (pDC2_sh_sgRNA1421800) was derived from the pDC2-Cas9-HDHFRyFCU plasmid [63] after removing the *hdhfr-yfcu* cassette by digestion with EcoRI followed by treatment with T4 DNA polymerase (NEB, n. M0203S) to generate blunt ends, digestion with HpaI and religation with T4 ligase (Roche, n. 10481220001). The sgRNA targeting the *PF3D7_1421800* coding region was prepared by annealing forward and reverse oligonucleotides and cloned into BpiI-digested plasmid using the InFusion system. Ring-stage 10E cultures were transfected with 15 µg of PvuI-linearized donor plasmid and 60 µg of circular Cas9 plasmid using a BioRad GenePulser Xcell electroporator. After transfection, cultures were selected for 4 days with 10 nM WR99210 (Jacobus Pharmaceuticals), and fresh RBCs were provided to the culture once per week until parasites were observed 15 days after transfection. Diagnostic PCR analysis of genomic DNA was used to confirm correct edition and absence of detectable parasites with wt *PF3D7_1421800* locus (S6A Fig). All primers used for PCR amplification of HRs, generation of sgRNAs and diagnostic PCR are described in S1 Table.

To knockout the *ap2-hs* gene from the K13^mut^ line using the CRISPR/Cas9 system (K13^mut^/Δ*ap2-hs* line), we used the same plasmids and approach previously described [15] (S8A Fig), with a modification in the quantity of plasmids used for transfection. In brief, ring stage cultures were transfected with 30 µg of circular pDC2_wo/hdhfr_ap2hs_sgRNA3’ plasmid, which encodes Cas9 and one sgRNA, and 60 µg of PvuI-linearized pL7-ap2hs_KO_sgRNA5’ donor plasmid, which contains the HRs flanking a *hdhfr* expression cassette and encodes a second sgRNA. After transfection using a BioRad GenePulser Xcell electroporator, selection with WR99210 and maintenance were performed as described above, and parasites were observed 13 days after transfection. Subclones were obtained by limiting dilution. Correct edition was confirmed by diagnostic PCR analysis of genomic DNA (S8A Fig) with primers described in S1 Table.

### HS assays and establishment of a new water bath-based standard HS assay

We previously used a cell culture incubator-based HS assay [15], but we found that it was difficult to achieve stable, consistent temperatures in an incubator, as others have previously reported [64]. Slight differences in the position of the cultures within the incubator, differences between incubator units, spontaneous temperature fluctuations and the number of times the incubator door was opened during the assay affected the results and compromised reproducibility. Therefore, we developed an alternative assay in which cultures are transferred to a centrifuge tube and HS is performed in a water bath, where temperature is more stable than in an incubator. Furthermore, the temperature of a water bath is easy to control and measure and corresponds well with the temperature of the cultures, whereas the temperature of an incubator does not immediately coincide with the temperature of the cultures within it.

For the new standard HS assays, cultures were synchronised to a 5 h age window by Percoll purification of late stages followed by sorbitol lysis (to remove late stages) 5 h later, resulting in a 0–5 hpi rings culture [65]. At 30–35 hpi, 4 ml of each culture at ~1.5% parasitaemia were transferred to 15 ml centrifuge tubes and gassed for 5 s with regular parasite culture gas mixture (5% CO_2,_ 2% O_2_, balance N_2_). The tubes were placed in a water bath at 41 ºC (HS samples) or at 37 ºC (control samples; 35 ºC for experiments with K13^mut^/Δ*ap2-hs* lines). The temperature of the water bath was calibrated using a precision glass thermometer and validated at each experiment. The tubes were placed in a plastic rack inside the water bath, avoiding the use of glass beakers to hold the tubes (we found that the temperature of the water inside a beaker was lower than in the rest of the water bath). After 1 h in the corresponding water baths, cultures were resuspended (typically by pipetting up and down) and two 100 µl aliquots of each sample were transferred to a 96-well plate and cultured for >30 h to assess HS survival by flow cytometry at the following cycle (at this time, all viable parasites had completed the IDC and reinvaded, despite the HS-induced delays in life cycle progression). The remaining volume of each sample (~4 ml) was centrifuged at 1,500 rpm for 5 min and the pellet resuspended in 20 pellet volumes (12 for rings) of TRIzol reagent (Invitrogen, n. 15596018) and stored at -80 ºC for transcriptional analysis. To measure parasitaemia by flow cytometry, 5 µl of culture were resuspended in 800 µl of PBS, stained with 1 µl of SYTO11 nucleic acid stain (Invitrogen, n. S7573) and incubated for 1 min before analysis in a BD FACSCalibur flow cytometer (BD Biosciences), as previously described [66]. The parasitaemia of cultures exposed to HS relative to the parasitaemia of control cultures was used to determine HS survival. Some dead parasites from the previous cycle (the cycle of HS) are still detected at the time of measuring parasitaemia (>30 h after HS), which can result in overestimation of survival rates when a substantial number of parasites die as a consequence of HS. However, light microscopy analysis of Giemsa-stained smears confirmed that under the standard HS conditions, even in the 10G line some parasites successfully reinvaded and generated new rings. For the incubator-based HS assay, 30–35 hpi cultures were exposed for 3 h to a 41.5ºC HS in a cell culture incubator, within an air-tight incubation chamber, as previously described [15].

Under the conditions of the new water bath-based standard HS assay, 1 h incubation at 41 ºC in a water bath, HS survival was similar to survival after a 3 h HS at 41.5 ºC in an incubator, our previous standard HS conditions [15]. The direct contact with water, which has higher thermal conductivity than air, results in cultures achieving the target temperature faster, explaining why a 1 h at 41 ºC HS in a water bath has an impact on parasite viability similar to a longer HS at a higher temperature in an incubator.

For the experiments to test the impact of different HS durations, one tube of each parasite line was placed in the water bath for each HS duration and, after the selected incubation time, each sample was taken from the water bath and processed as mentioned above. For the experiments to test the impact of different HS temperatures, six different water baths at different temperature were used to simultaneously expose identical aliquots of the same synchronised cultures to the different temperatures. To assess the effect of HS at different stages of the IDC, HS was applied to cultures at different points of the IDC derived from the same tightly synchronised culture.

### Flow cytometry analysis of parasite growth and viability after HS

30-35 hpi cultures exposed to a standard 1 h at 41 ºC HS (or control cultures incubated in parallel in a water bath at 37 ºC) were analysed at different times after HS by flow cytometry. Samples were stained with Hoechst 33342 (Sigma-Aldrich, n. 14533) to measure DNA content and with MitoTracker Deep Red FM (Invitrogen, n. M22426) to assess mitochondrial membrane potential as a proxy for viability. Staining with MitoTracker at a final concentration of 0.3 µM was performed as previously described [40], but in combination with 5 µg/ml Hoescht instead of SYTO 11. Flow cytometry analysis was performed with a CytoFLEX LX cytometer (Beckman Coulter), using a gating strategy similar to the one previously described [40] (S2A Fig), to measure Hoechst (near UV laser: 375 nm; filter: 450/45; power: 57 mW) and MitoTracker (red laser: 638 nm; filter: 660/10; power: 52 mW) fluorescence. The analysis was first performed 3 h after HS (34–39 hpi). At this point, the cyclic GMP-dependent protein kinase inhibitor ML10 [42] was added to the remaining culture (80 nM), to prevent schizont rupture. Cultures were again analysed by flow cytometry 12 h and 15 h after ML10 addition (46–51 hpi and 49–54 hpi, respectively).

### RNA extraction and RT-qPCR

RNA was collected in TRIzol immediately after HS or drug treatment and purified using a method suitable for low amounts of RNA [67]. In brief, RNA was purified using the RNeasy Mini Kit (Qiagen, n. 74104), DNase treated using the RNase-free DNase set (Qiagen, n. 79254) and cDNA prepared by reverse transcription using the AMV Reverse Transcription Kit (Promega, n. A3500), as previously described [65]. The resulting cDNAs were used to quantify transcript abundance by qPCR, using the PowerSYBR Green master mix (Applied Biosystems, n. 4368708) and the standard curve method, with a standard curve included in each plate for each primer pair [65]. Each sample was tested in triplicate and negative controls (reactions without reverse transcriptase) tested in duplicate for a selection of the genes analysed. Transcript levels were normalised using *serine-tRNA ligase* (*serrs*, *PF3D7_0717700*) or *ubiquitin-conjugating enzyme* (*uce*, *PF3D7_0812600*). Primers used for qPCR are described in S1 Table. The Ct values from the qPCR analyses are presented in S1 Data.

### Gametocyte experiments

To induce sexual conversion in the E5ind line, cultures were sorbitol-synchronised and ~20 h later treated with rapamycin for 1 h to induce DiCre-mediated recombination [47]. The next day, cultures were placed in complete medium supplemented with 10% B^+^ human serum instead of Albumax II, to support gametocyte development, and heparin (Merck, H3149-50KU) was added to remove asexual parasites (20 U/ml) [68]. The culture medium (with human serum and heparin) was changed daily throughout the experiment. A standard HS was performed on day 2 (stage I) or on day 4 (stage II) after rapamycin treatment. To determine HS survival, 2 days after HS the gametocytaemia of cultures exposed to HS and control cultures maintained in parallel at 37 ºC was measured by light microscopy analysis of Giemsa-stained smears (pyknotic parasites not counted). In all experiments, in cultures exposed to HS we observed reduced gametocytaemia (relative to controls), rather than major alterations in gametocyte stage composition. This indicates that HS resulted in death and disintegration or collapse of sensitive gametocytes, rather than just developmental arrest.

The NF54-*gexp02*-Tom line was regularly maintained under shaking conditions in culture medium supplemented with 2 mM choline to prevent sexual conversion. To allow sexual conversion of a large proportion of the parasites, cultures were sorbitol-synchronised and then maintained in medium without choline for 48 h under static conditions [48]. After 48 h, Albumax II-supplemented medium was replaced by medium supplemented with B^+^ human serum or with Albumax II plus 1x ITS-X (Gibco, n. 51500056), which is a recently described human serum alternative to support full development of viable gametocytes [69]. While initial experiments were performed with human serum-supplemented medium, we found that ITS-X was much easier to obtain and prepare and improved gametocyte development, so we used it for the remaining experiments. We did not observe differences in the results of the experiments between cultures supplemented with human serum or with ITS-X. At 48 h after choline depletion, we also added heparin to the culture medium to remove asexual forms. Medium was changed daily for the next 4 days and then every 2 or 3 days until the end of the experiment. NF54-*gexp02*-Tom gametocyte cultures were exposed to a standard HS at day 3 after adding heparin (stage II of sexual development) or between days 10 and 14, when the vast majority of gametocytes had reached full maturity (stage V). HS survival was determined as described above for the E5ind line, by light microscopy analysis of Giemsa-stained smears prepared 2 days after HS.

Exflagellation assays [70] were performed with mature gametocyte cultures 2.5-5 h after HS, typically on day 13 after adding heparin. 30 µl of cultures exposed to HS and their controls were placed in 1.5 ml tubes at 37 ºC. Cultures were spun and 10 µl of the supernatant removed. Next, gamete activation was induced by diluting 1:2 with ookinete media (standard RPMI-HEPES with hypoxanthine, NaHCO_3_ and 100 µM xanthurenic acid) and incubating at room temperature. For each sample, ~ 10 µl were loaded to a haemocytometer and, 12 min after inducing activation, exflagellation centres were counted by light microscopy with a 40X objective. Each culture was tested twice (two technical replicates). To calculate the percentage of gametocytes that exflagellated, we used the number of exflagellation centres per µl determined with the haemocytometer, and the number of gametocytes per µl, which was estimated from the number of RBCs per µl (determined in the same haemocytometer) and the gametocytaemia of the culture (determined from Giemsa-stained smears prepared immediately before the assay).

Gamete activation and egress assays were performed with mature gametocyte cultures 2.5-5 h after HS, typically at day 14 after adding heparin, following a previously described protocol [71]. After Percoll purification to enrich for gametocytes, parasites from cultures exposed to HS or control cultures were resuspended in 150 µl of complete culture medium (Albumax II and ITS-X-supplemented) and stained with WGA-Oregon Green 488 conjugate (Invitrogen, n. W6748) at a final concentration of 2.5 µg/ml and Hoechst 33342 (Sigma-Aldrich, n. 14533) at a final concentration of 2 µg/ml. After 15 min at 37 ºC, samples were washed with RPMI-HEPES (at the same concentration as in the parasite culture medium), followed by resuspension in culture medium with 20 µM xanthurenic acid and ≥15 min incubation at room temperature to induce gamete activation and egress. After spinning and resuspending in PBS, wet preparations were inspected in a fluorescence microscope. Parasites were scored as gametocytes (elongated and with peripheral WGA-488 signal), activated gametes (rounded-up but not egressed from RBCs, *i.e.*, with peripheral WGA-488 signal) or activated and egressed gametes (absence of WGA-488 signal).

### DHA and CQ sensitivity assays

Cultures were synchronised to a 5 h age window using the Percoll-sorbitol method, as described above, and adjusted to a 1% (experiments with Dd2-derived lines) or 1.5% (experiments with 10E and 10G) parasitaemia. At 30–35 hpi, cultures were exposed to different concentrations of DHA (Sigma-Aldrich D7439) or CQ (Sigma-Aldrich C6628) for 3 h. Immediately after removing DHA or CQ, ~ 4 ml of each sample were collected in TRIzol for RNA extraction (as described above; only 10E and 10G experiments) and, from the rest of the sample, 100 µl (in culture medium without drug) were transferred in duplicate or triplicate to a 96-well plate and placed under culture again. At the following cycle, ~ 60–65 h after synchronisation, Giemsa-stained smears were prepared for each sample in duplicate or triplicate. Parasitaemias were determined by light microscopy inspection of the smears and parasite survival calculated relative to the parasitaemia of the control (untreated) culture. For IC_50_ determinations, drug concentrations were log_10_-transformed, and percent survival values were fit to a dose-response sigmoidal curve.

### Ring Survival Assay (RSA)

RSAs were performed according to a standard protocol [51]. Cultures of the Dd2, K13^mut^ and K13^mut^/Δ*ap2-hs* (6B and 12C subclones) parasite lines were synchronised to a 3 h age window using the Percoll-sorbitol method and adjusted to 1% parasitaemia. Immediately after synchronisation, at 0–3 hpi, a fraction of each culture (1.6 ml) was exposed to either a physiological dose of DHA (700 nM final concentration) or dimethyl sulfoxide (DMSO) carrier (1% final concentration) as a control, and incubated for 6 h at 35 ºC. Next, 1 ml of each culture was collected, resuspended in TRIzol and stored at -80 ºC for future RNA extraction. DHA was removed from the remaining culture and, after washing with RPMI-HEPES, 100 µl of each culture were transferred in triplicate to a 96-well plate and cultured for 66 h at 35 ºC. At this time, Giemsa-stained smears were prepared for each sample in duplicate/triplicate and parasitaemias determined by light microscopy.

### Statistical analysis

Statistical analysis was performed using GraphPad Prism 10.6.1. To determine the statistical significance of differences between parasite lines, stages or conditions, unless otherwise stated, we used two-tailed unpaired Student’s *t*-tests for pairwise comparisons and, for multiple comparisons, unpaired one-way ANOVA with Tukey’s correction for multiple testing. For experiments in which activation of the HSR and survival were studied under different conditions (*e.g.*, different HS duration, temperature or stage of exposure), the statistical analysis focused on determining whether or not the HSR was activated and enhanced survival. Therefore, for each condition, we compared activation of the expression of HSR genes or survival between a parasite line with wild-type AP2-HS (10E) and a parasite line with a mutation in AP2-HS that prevents activation of the HSR (10G), using pairwise comparisons.

## Supporting information

S1 Fig Transcript levels of *hsp70–1* and *hsp90* after HS of different duration or at different temperatures. (PDF)

S2 Fig Flow cytometry and microscopy analysis of cultures after exposure to HS. (PDF)

S3 Fig Transcriptional changes after HS at different stages of the IDC using *uce* as a normalising gene. (PDF)

S4 Fig Analysis of the effect of HS on gametocytes. (PDF)

S5 Fig Changes in *PF3D7_1421800* transcript levels after HS with different conditions. (PDF)

S6 Fig Generation and characterisation of the *PF3D7_1421800* KO line. (PDF)

S7 Fig Transcriptional changes after exposure to a DHA or CQ pulse. (PDF)

S8 Fig Generation and characterisation of K13 and AP2-HS mutant lines. (PDF)

S9 Fig Transcriptional changes in K13 and AP2-HS mutant lines in the ring survival assay (RSA). (PDF)

S1 Table Primers used in this study. (PDF)

S1 Data Ct values for the qPCR analyses. (XLSX)
