# Supplementary material for: Characterisation of the thermal and non-thermal stress conditions that activate the Plasmodium falciparum AP2-HS-dependent heat-shock response
Source: PLoS Pathog. 2026 Jul 9;22(7):e1014346. doi: 10.1371/journal.ppat.1014346 (PMC13349141; doi:10.1371/journal.ppat.1014346)
Supplement: S1 Fig — (PDF) [file ppat.1014346.s001.pdf]

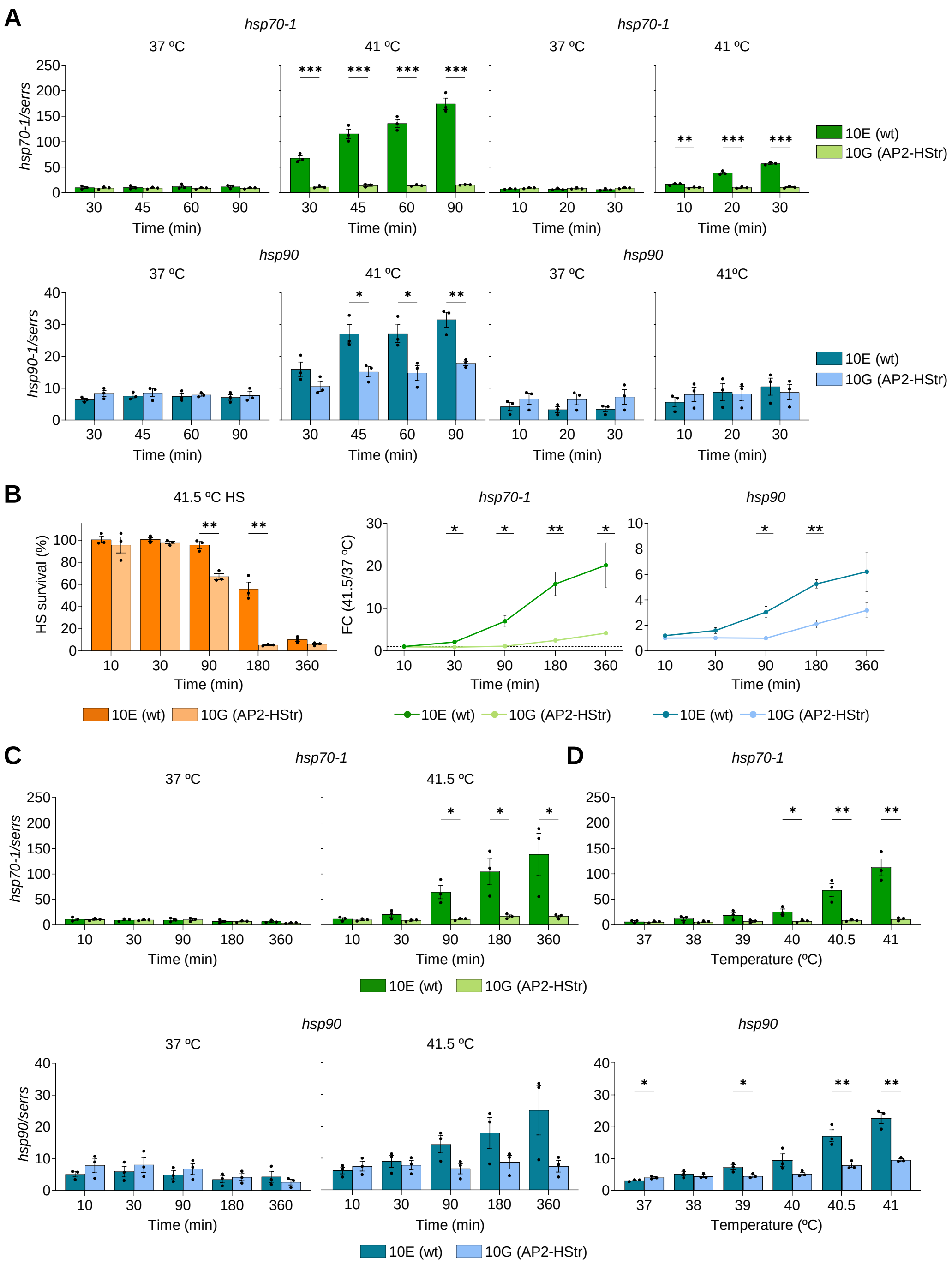

S1 Fig. Transcript levels of *hsp70-1* and *hsp90* after HS of different duration or at different temperatures. A. Transcript levels of *hsp70-1* and *hsp90*, normalised against *serrs* transcripts, in cultures exposed to a HS of variable duration (41 ºC) or not (37 ºC) in a water bath. **B.** Left, HS survival after exposing cultures to a HS of variable duration at 41.5 ºC in an incubator, relative to control cultures (no HS). Right, fold-change (FC) of *serrs*-normalised *hsp70-1* and *hsp90* transcript levels in cultures exposed to HS relative to controls (no HS). The horizontal dotted line indicates a FC of 1 (no change). **C.** Transcript levels of *hsp70-1* and *hsp90*, normalised against *serrs* transcripts, for the same samples as in panel B. **D.** Transcript levels of *hsp70-1* and *hsp90*, normalised against *serrs* transcripts, in cultures exposed to HS for 1 h at variable temperature in a water

bath or control cultures (37 ºC). In all panels, values are the mean ± s.e.m. of n=3 independent biological replicates. Statistically-significant differences between 10E and 10G, calculated using two-sided unpaired Student's *t*-tests, are indicated by asterisks (*: $0.01<P\leq0.05$; **: $0.001<P\leq0.01$; ***: $P\leq0.001$).
