# Supplementary material for: Characterisation of the thermal and non-thermal stress conditions that activate the Plasmodium falciparum AP2-HS-dependent heat-shock response
Source: PLoS Pathog. 2026 Jul 9;22(7):e1014346. doi: 10.1371/journal.ppat.1014346 (PMC13349141; doi:10.1371/journal.ppat.1014346)
Supplement: S2 Fig — (PDF) [file ppat.1014346.s002.pdf]

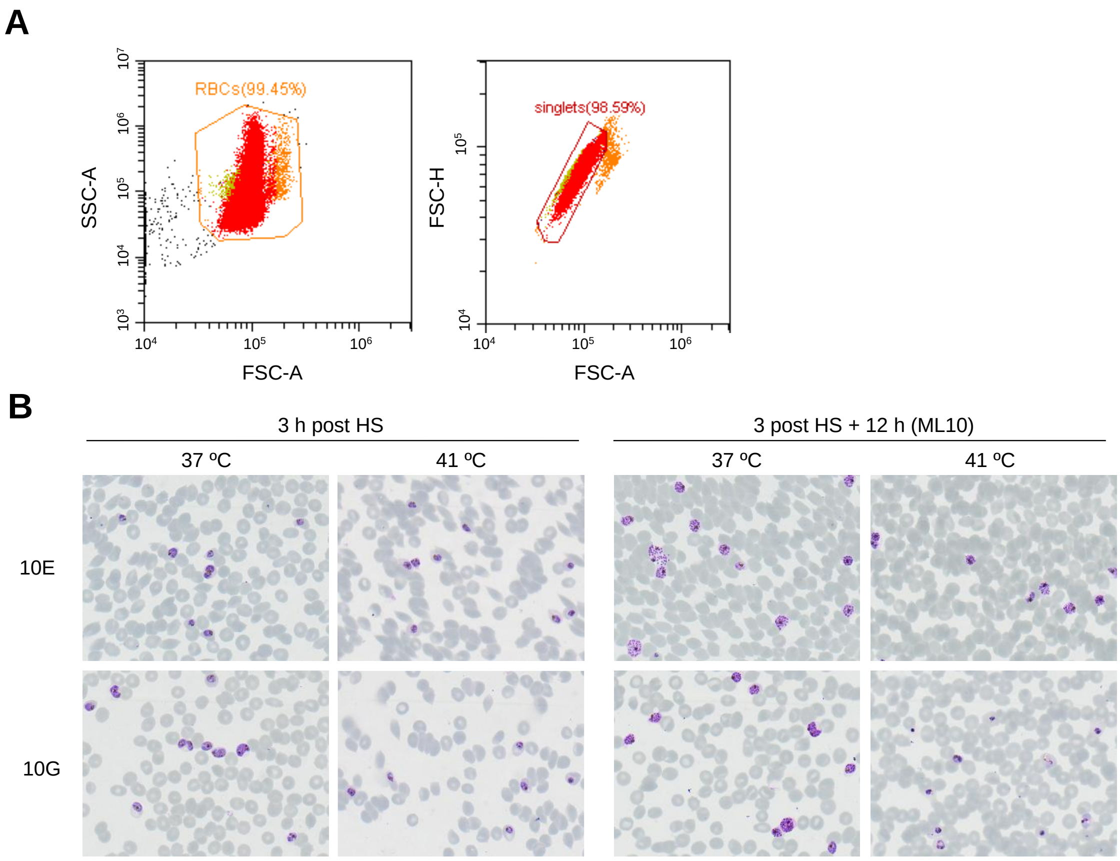

S2 Fig. Flow cytometry and microscopy analysis of cultures after exposure to HS. A. Representative scatter plots of the initial gating strategy for the flow cytometry analysis of cultures after exposure to HS. Red blood cells (RBCs) were first gated based on SSC-A vs FSC-A plots. Next, singlets were identified based on FSC-H vs FSC-A plots. All downstream analyses were done using only singlets. **B.** Representative light microscopy images of Giemsa-stained smears of 10E and 10G cultures exposed to a HS at 41 ºC for 1 h or control cultures (37 ºC), prepared 3 h post exposure, or after additional incubation for 12 h (with ML10 to prevent schizont bursting).
