# Supplementary material for: Characterisation of the thermal and non-thermal stress conditions that activate the Plasmodium falciparum AP2-HS-dependent heat-shock response
Source: PLoS Pathog. 2026 Jul 9;22(7):e1014346. doi: 10.1371/journal.ppat.1014346 (PMC13349141; doi:10.1371/journal.ppat.1014346)
Supplement: S3 Fig — (PDF) [file ppat.1014346.s003.pdf]

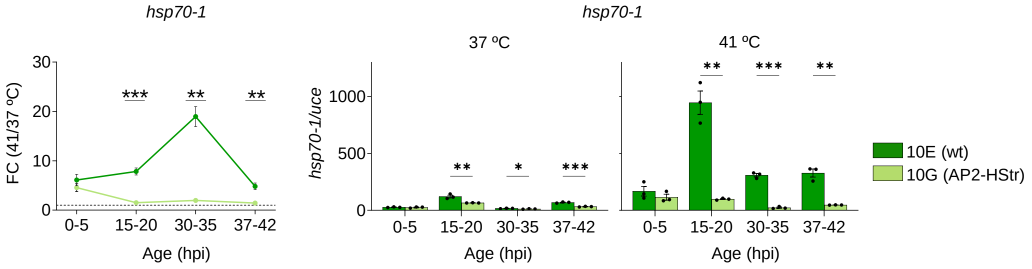

S3 Fig. Transcriptional changes after HS at different stages of the IDC using *uce* as a normalising gene. Left, fold-change (FC) of *uce*-normalised *hsp70-1* transcript levels in cultures at different stages of the IDC exposed to a HS at 41 ºC for 1 h, relative to transcript levels in control cultures (no HS). Right, *uce*-normalised *hsp70-1* transcript levels in cultures at different stages of the IDC exposed to HS (41 ºC) or not (37 ºC). In all panels, values are the mean ± s.e.m. of n=3 independent biological replicates. Statistically-significant differences between 10E and 10G, calculated using two-sided unpaired Student's *t*-tests, are indicated by asterisks (*: 0.01<*P*≤0.05; **: 0.001<*P*≤0.01; ***: *P*≤0.001).
