# Supplementary material for: Characterisation of the thermal and non-thermal stress conditions that activate the Plasmodium falciparum AP2-HS-dependent heat-shock response
Source: PLoS Pathog. 2026 Jul 9;22(7):e1014346. doi: 10.1371/journal.ppat.1014346 (PMC13349141; doi:10.1371/journal.ppat.1014346)
Supplement: S4 Fig — (PDF) [file ppat.1014346.s004.pdf]

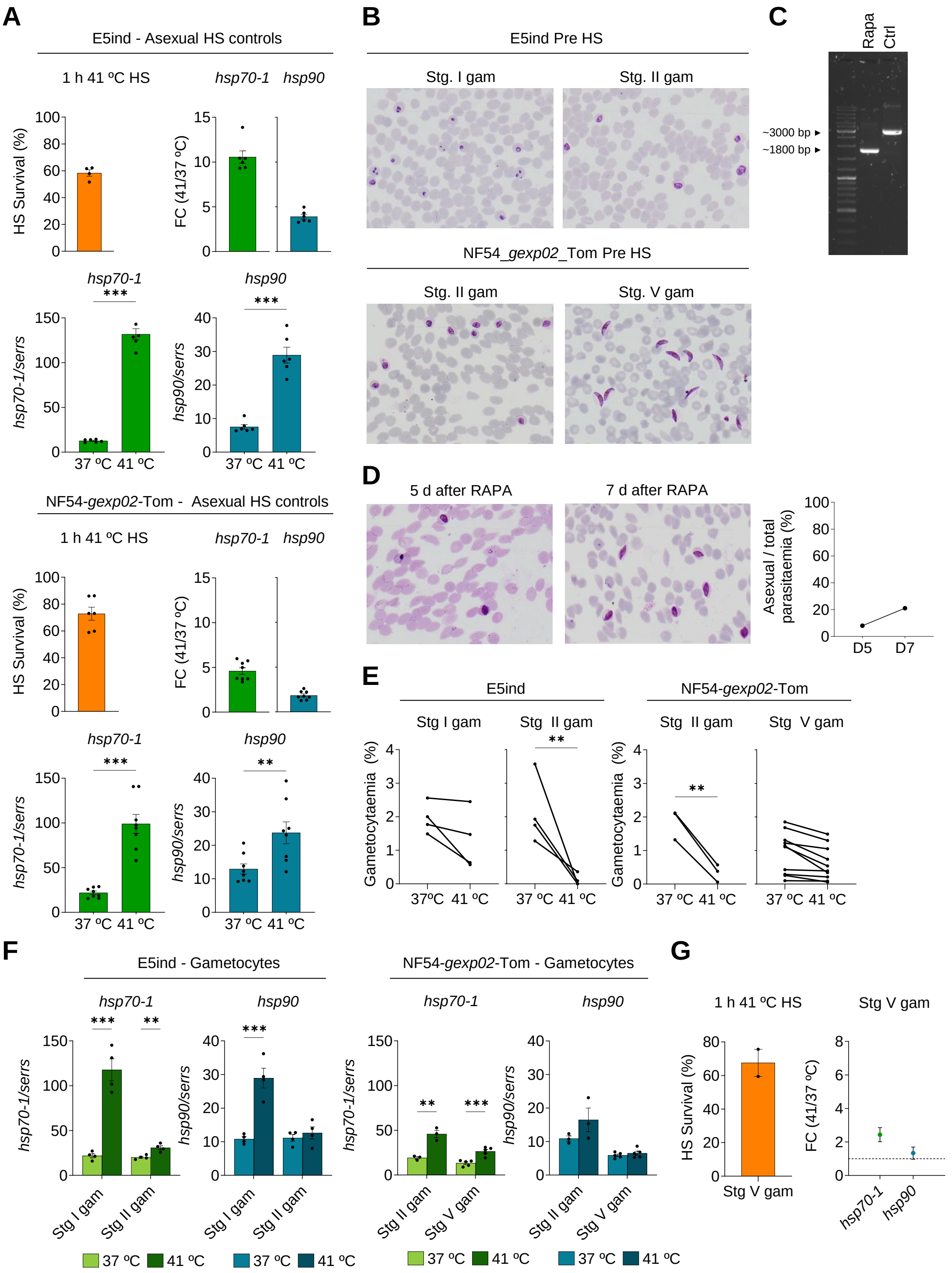

S4 Fig. Analysis of the effect of HS on gametocytes. A. Analysis of HS survival, fold-change (FC) of *serrs*-normalised *hsp70-1* and *hsp90* transcript levels (cultures exposed to HS relative to control cultures) and *serrs*-normalised transcript levels of *hsp70-1* and *hsp90* for E5ind and NF54-*gexp02*-Tom asexual parasite cultures (containing mainly late trophozoite and early schizont stages) exposed to a HS at 41 ºC for 1 h and controls (no HS). Values are the mean ± s.e.m. of n=4 to 8 independent biological replicates. **B.** Representative images of

Giemsa-stained smears of gametocyte cultures just before exposing them to HS (pre HS) at different stages. **C.** Diagnostic PCR analysis of E5ind genomic DNA to assess recombination at the *ap2-g* locus ~20 h after adding Rapamycin (Rapa) in induced cultures, or adding only the DMSO vehicle in control (Ctrl) cultures. The expected size of the bands for correct recombination (~1,800 bp) or absence of recombination (~3,000 bp) is indicated. **D.** Light microscopy analysis of Giemsa-stained smears from E5ind cultures prepared 5 or 7 days after adding Rapamycin and maintained without heparin, and quantification of the proportion of asexual parasites (mainly ring stages) at days 5 or 7 (n=1). **E.** Gametocytaemia of cultures exposed to HS (41 ºC) or not (37 ºC), measured two days after exposure. Lines connect values for the same culture. **F.** Transcript levels of *hsp70-1* and *hsp90*, normalised against *serrs* transcripts, in E5ind or NF54-*gexp02*-Tom cultures at different stages of gametocyte development exposed to a 1 h HS (41 ºC) or not (37 ºC). Values are the mean ± s.e.m. of n=3 to 5 independent biological replicates. **G.** HS survival and fold-change (FC) of *serrs*-normalised *hsp70*-1 and *hsp90* transcript levels in NF54-*gexp02*-Tom mature (stage V) gametocyte cultures exposed to a 1h HS at 41ºC, relative to control cultures (no HS). These data correspond to the gametocyte cultures used for egress and exflagellation assays and is analogous to experiments presented in main Fig. 4E. Values are the mean ± s.e.m of n=2 independent biological replicates, with each value for HS survival corresponding to the mean of two technical replicates (independent experiments performed on the same cultures) for each biological replicate. In panels A, E and F, statistically-significant differences between HS and no-HS cultures, calculated using two-sided unpaired Student's *t*-tests, are indicated by asterisks (*: $0.01<P\leq0.05$; **: $0.001<P\leq0.01$; ***: $P\leq0.001$).
