# Supplementary material for: Characterisation of the thermal and non-thermal stress conditions that activate the Plasmodium falciparum AP2-HS-dependent heat-shock response
Source: PLoS Pathog. 2026 Jul 9;22(7):e1014346. doi: 10.1371/journal.ppat.1014346 (PMC13349141; doi:10.1371/journal.ppat.1014346)
Supplement: S5 Fig — (PDF) [file ppat.1014346.s005.pdf]

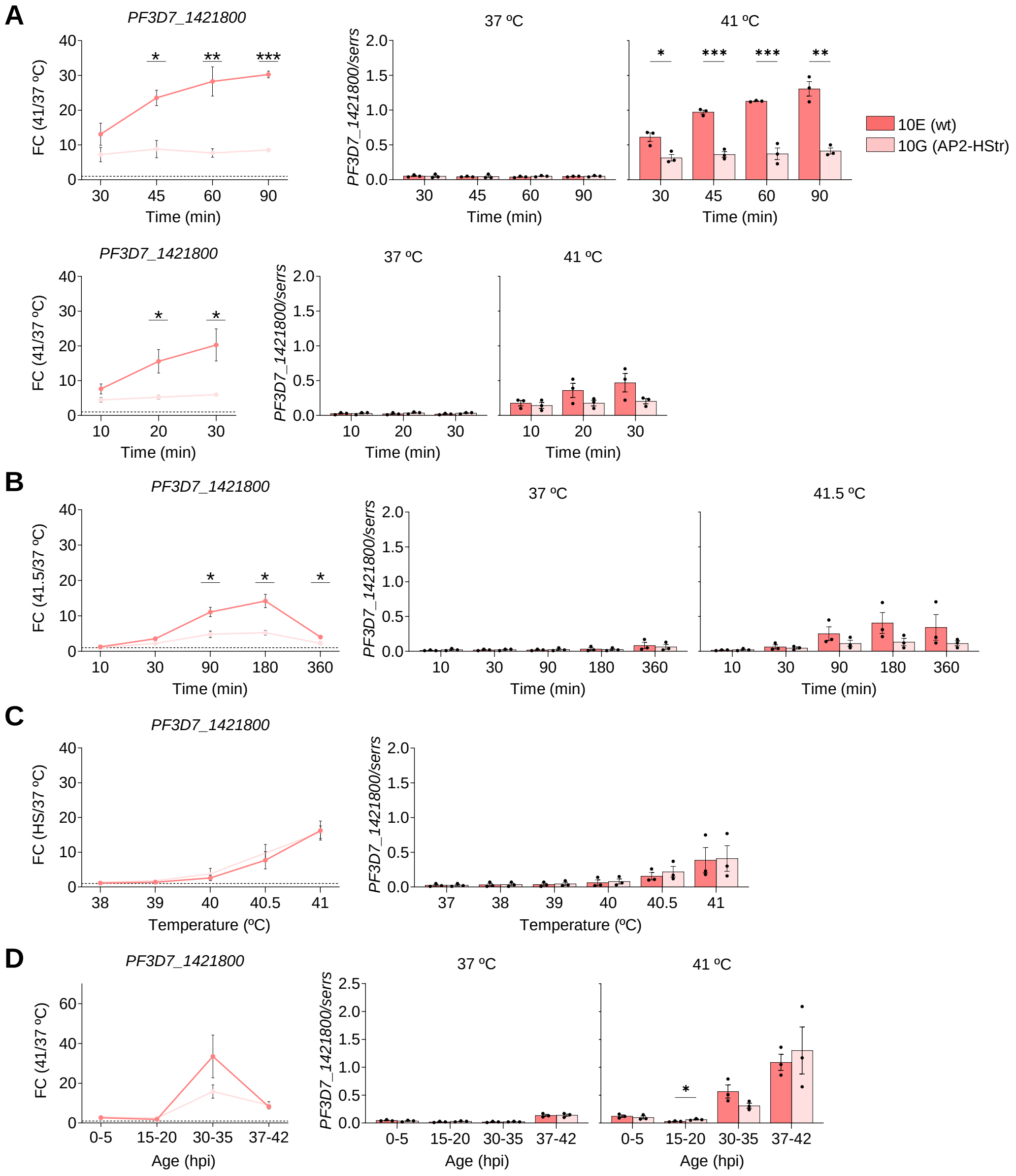

S5 Fig. Changes in *Pf3D7_1421800* transcript levels after HS with different conditions. A. Left, fold-change (FC) of *serrs*-normalised *Pf3D7_1421800* transcript levels in 10E (wt) and 10G (AP2-HStr) cultures exposed to a 41 ºC HS of variable duration in a water bath relative to transcript levels in control cultures (no HS). The horizontal dotted line indicates a FC of 1 (no change). Right, transcript levels of *PF3D7_14241800*, normalised against *serrs* transcripts, in cultures exposed to a HS of variable duration (41 ºC) or not (37 ºC). **B.** Same as in panel A, for experiments performed in an incubator. **C.** Same as in panel A, for experiments with HS for 1 h at variable temperature. **D.** Same as in panel A, for experiments in which cultures were exposed to a HS at 41 ºC for 1 h at different stages of the IDC. In all panels, values are the mean ± s.e.m. of n=3 independent biological replicates. Statistically-significant differences between 10E and 10G, calculated using two-sided unpaired Student's *t*-tests, are indicated by asterisks (*: 0.01<*P*≤0.05; **: 0.001<*P*≤0.01; ***: *P*≤0.001).
