# Supplementary material for: Characterisation of the thermal and non-thermal stress conditions that activate the Plasmodium falciparum AP2-HS-dependent heat-shock response
Source: PLoS Pathog. 2026 Jul 9;22(7):e1014346. doi: 10.1371/journal.ppat.1014346 (PMC13349141; doi:10.1371/journal.ppat.1014346)
Supplement: S6 Fig — (PDF) [file ppat.1014346.s006.pdf]

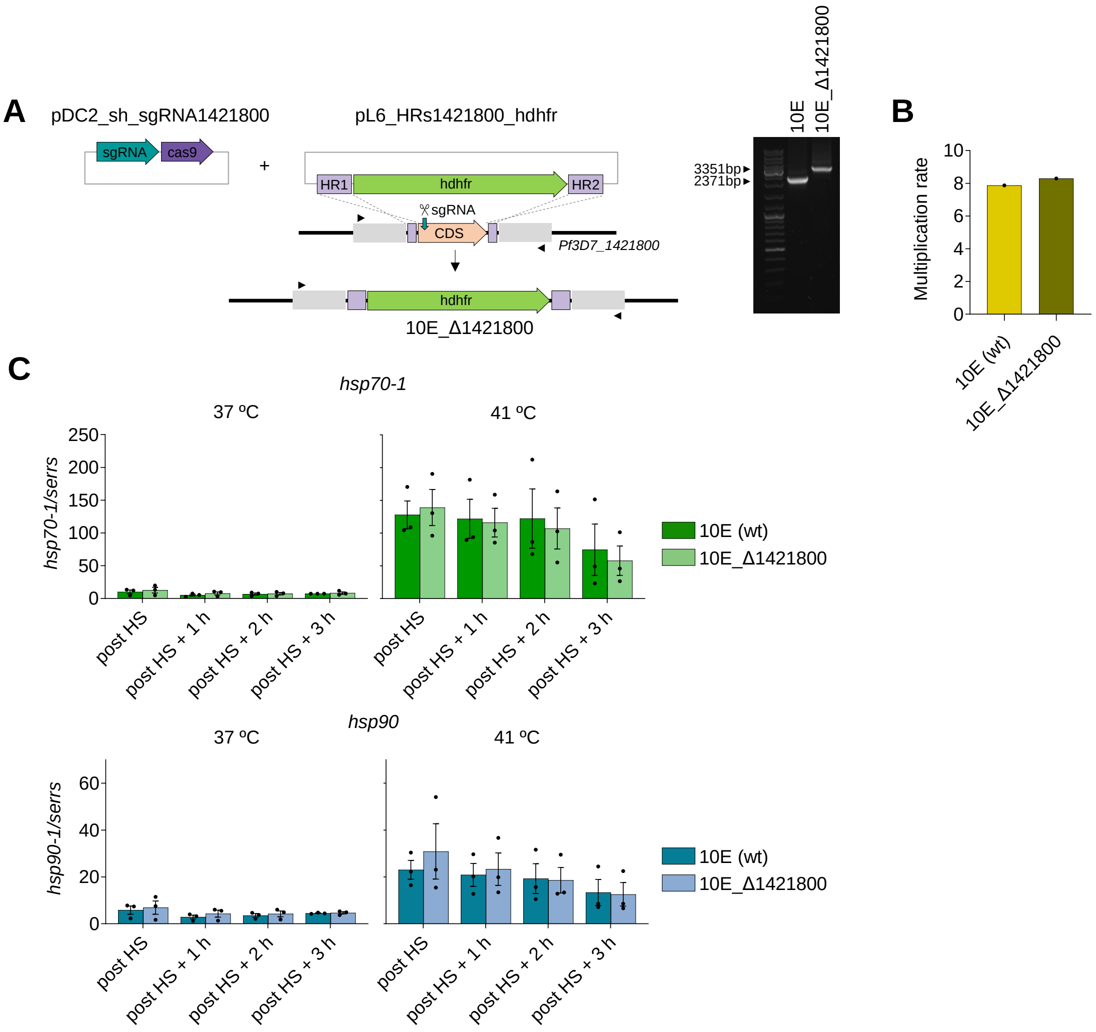

S6 Fig. Generation and characterisation of the *PF3D7_1421800* KO line. A. Schematic of the CRISPR-Cas9 strategy to knockout the *PF3D7_1421800* gene and diagnostic PCR analysis of the *PF3D7_1421800* locus showing correct editing. PCR was performed with primers (arrowheads) external to the homology regions (HRs) (Table S1, primers 12/13). The position targeted for cleavage by the guide RNA (sgRNA) is indicated. **B.** Multiplication rate of 10E and 10E_Δ1421800, determined by flow-cytometry measures of parasitaemia at two consecutive cycles. Data from n=1 biological replicate. **C.** Transcript levels of *hsp70-1* and *hsp90*, normalised against *serrs* transcripts, in 10E (wt) and 10E_Δ1421800 cultures immediately after a standard 1 h HS at 41 ºC (post HS) or after additional incubation at 37ºC. Values are the mean ± s.e.m. of n=3 independent biological replicates. No statistically significant differences were observed at any time point between 10E and 10E_Δ1421800, using a two-sided unpaired Student’s *t*-test.
