# Supplementary material for: Characterisation of the thermal and non-thermal stress conditions that activate the Plasmodium falciparum AP2-HS-dependent heat-shock response
Source: PLoS Pathog. 2026 Jul 9;22(7):e1014346. doi: 10.1371/journal.ppat.1014346 (PMC13349141; doi:10.1371/journal.ppat.1014346)
Supplement: S7 Fig — (PDF) [file ppat.1014346.s007.pdf]

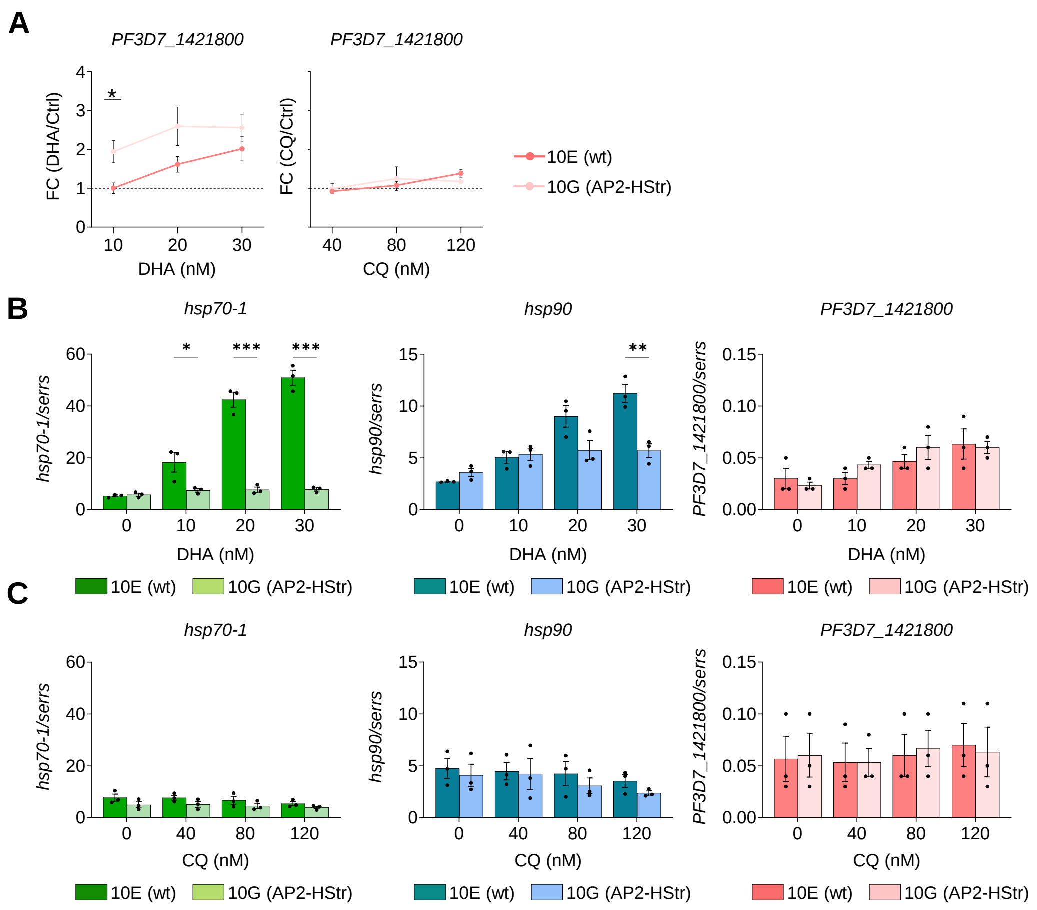

S7 Fig. Transcriptional changes after exposure to a DHA or CQ pulse. A. Fold-change (FC) of *serrs*-normalized *PF3D7_1421800* transcript levels in 10E (wt) and 10G (AP2-HStr) cultures exposed to a 3 h DHA or CQ pulse at different concentrations, relative to controls not exposed to drugs. The horizontal dotted line indicates a FC of 1 (no change). **B.** Transcript levels of *hsp70-1*, *hsp90*, and *PF3D7_1421800*, normalised against *serrs* transcripts, in 10E (wt) and 10G (AP2-HStr) cultures exposed to a 3 h DHA pulse at different concentrations. **C.** Same as panel B, for cultures exposed to CQ instead of DHA. In all panels, values are the mean ± s.e.m. of n=3 independent biological replicates. Statistically-significant differences between 10E and 10G, calculated using two-sided unpaired Student's *t*-tests, are indicated by asterisks (*: $0.01<P\leq0.05$; **: $0.001<P\leq0.01$; ***: $P\leq0.001$).
