# Supplementary material for: Characterisation of the thermal and non-thermal stress conditions that activate the Plasmodium falciparum AP2-HS-dependent heat-shock response
Source: PLoS Pathog. 2026 Jul 9;22(7):e1014346. doi: 10.1371/journal.ppat.1014346 (PMC13349141; doi:10.1371/journal.ppat.1014346)
Supplement: S8 Fig — (PDF) [file ppat.1014346.s008.pdf]

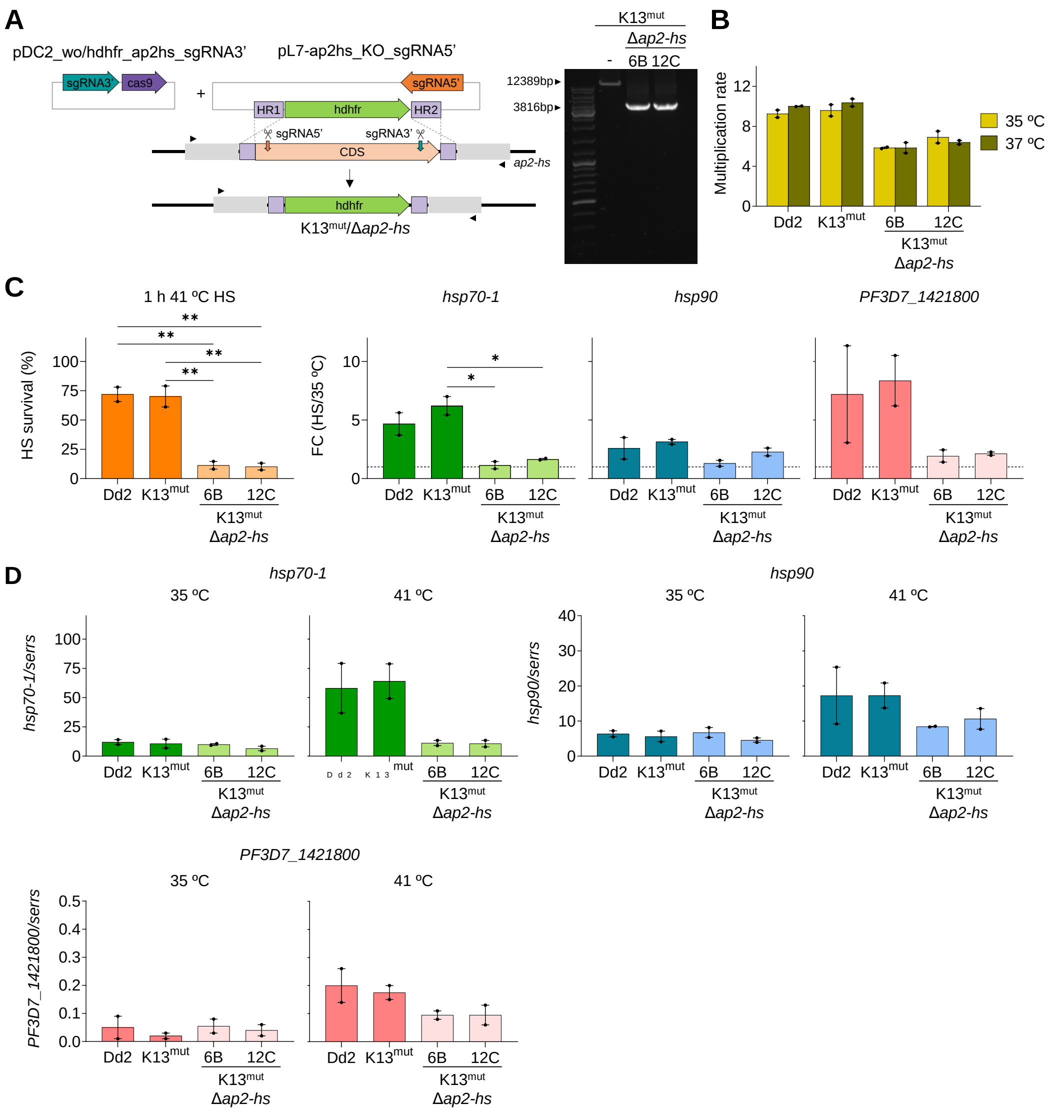

S8 Fig. Generation and characterisation of K13 and AP2-HS mutant lines. **A.** Schematic of the CRISPR-Cas9 strategy to knockout the *ap2-hs* gene and diagnostic PCR analysis of the *ap2-hs* locus to validate correct editing. PCR was performed with primers (arrowheads) external to the homology regions (HRs) (Table S1, primers 1/2). The positions targeted for cleavage by the guide RNAs (sgRNAs) are indicated. **B.** Multiplication rate of Dd2 (wt), K13 and AP2-HS mutants at 35 ºC and 37 ºC, determined by flow-cytometry measures of parasitaemia at two consecutive cycles. Values are the mean ± s.e.m. of n=2 independent biological replicates. **C.** Left, HS survival after exposing 30-35 hpi cultures of Dd2 (wt) and mutant parasite lines to a 1 h HS at 41 ºC, relative to control cultures not exposed to HS. Right, fold-change (FC) of *serrs*-normalised *hsp70-1*, *hsp90,* and *PF3D7_1421800* transcript levels in wt and mutant cultures exposed to HS relative to transcript levels in control cultures (no HS). **D.** Transcript levels of *hsp70-1*, *hsp90* and *PF3D7_1421800*, normalised against *serrs* transcripts, in cultures exposed to HS (41 ºC) or not (35 ºC). In all panels, values are the mean ± s.e.m. of n=3 independent biological replicates.

Statistically-significant differences between strains, calculated using one-way ANOVA, are indicated by asterisks (*: $0.01<P\leq0.05$; **: $0.001<P\leq0.01$; ***: $P\leq0.001$).
