# Supplementary material for: Characterisation of the thermal and non-thermal stress conditions that activate the Plasmodium falciparum AP2-HS-dependent heat-shock response
Source: PLoS Pathog. 2026 Jul 9;22(7):e1014346. doi: 10.1371/journal.ppat.1014346 (PMC13349141; doi:10.1371/journal.ppat.1014346)
Supplement: S9 Fig — (PDF) [file ppat.1014346.s009.pdf]

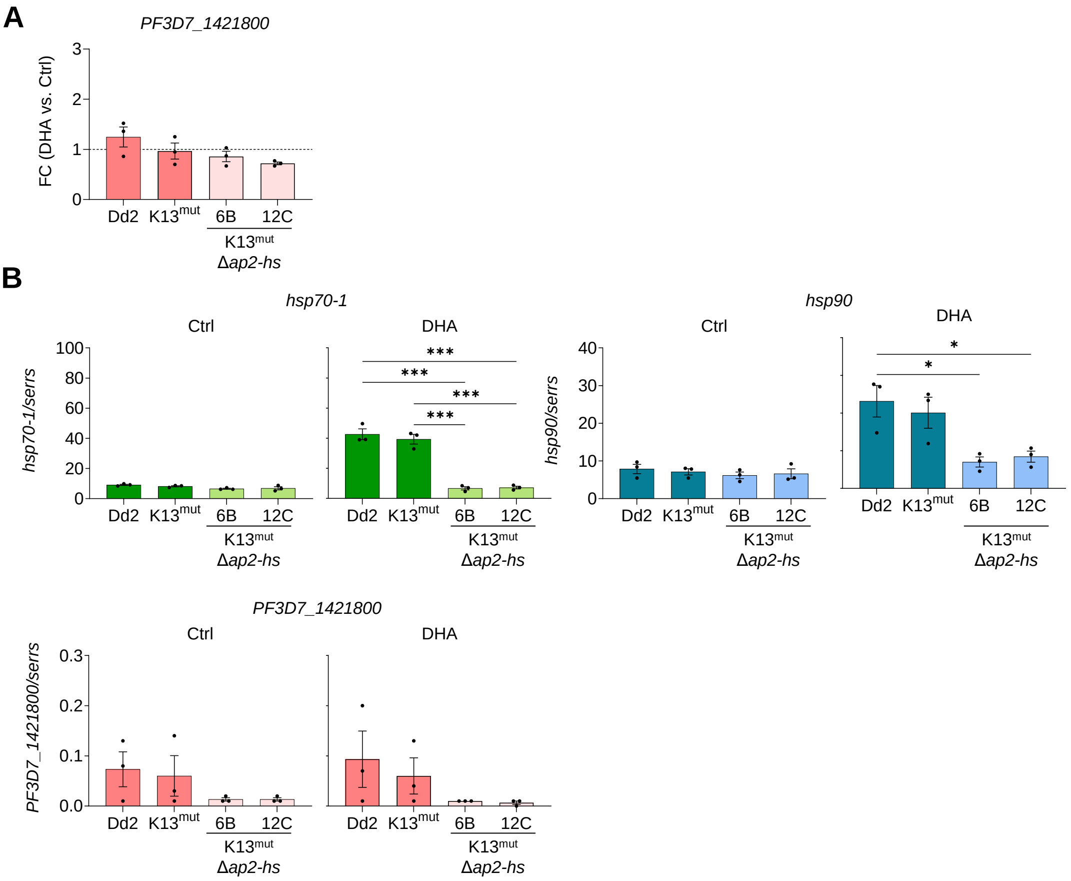

S9 Fig. Transcriptional changes in K13 and AP2-HS mutant lines in the ring survival assay (RSA). A. Fold-change (FC) of *serrs*-normalized *PF3D7_1421800* transcript levels in Dd2 wt and mutant cultures exposed to a 6 h 700 nM DHA pulse at 0-3 hpi in the RSA, relative to transcript levels in control cultures (DMSO vehicle only, no DHA). **B.** Transcript levels of *hsp70-1*, *hsp90*, and *PF3D7_1421800*, normalised against *serrs* transcripts, in cultures exposed to a DHA pulse in the RSA or not (Ctrl). In all panels, values are the mean ± s.e.m. of n=3 independent biological replicates. Statistically-significant differences between parasite lines, calculated using one-way ANOVA, are indicated by asterisks (*: 0.01<P≤0.05; **: 0.001<P≤0.01; ***: P≤0.001).
