# Supplementary material for: Characterisation of the thermal and non-thermal stress conditions that activate the Plasmodium falciparum AP2-HS-dependent heat-shock response
Source: PLoS Pathog. 2026 Jul 9;22(7):e1014346. doi: 10.1371/journal.ppat.1014346 (PMC13349141; doi:10.1371/journal.ppat.1014346)
Supplement: S1 Table — (PDF) [file ppat.1014346.s010.pdf]

	Name	Sequence (5' → 3')	Use
1	ap2hs_-94_Fw	CAGTTGATGATTACATCTCTG	Ext. PCR *ap2hs* KO Fw
2	ap2hs_end_+718_Rv	TTCATCACTTGTTAAGCATCC	Ext. PCR *ap2hs* KO Rv
3	ap2hs_+10399_Fw	AGAAATGAACAAAACATATTGGG	Copy nº qPCR *ap2hs* KO Fw
4	ap2hs_+10510_Rv	TTATATTTGTTATAGGTTCCTCC	Copy nº qPCR *ap2hs* KO Rv
5	1421800_-460_Fw_InF	gccggggaggactagTATTGTGTACTTTTCCAATGAAG	Amp. HR1 1421800 Fw
6	1421800_+2_Rv	TCCTTCCTCTACTTTTACATTC	Amp. ext HR1 1421800 Rv
7	1421800_-25_Rv_InF	ttacaaaatgcttaagATTGTATTTTTATTTTATAATTTAA	Amp. HR1 1421800 Rv
8	1421800_+1116_Fw_InF	attaaatctagaattcTTTAATGAAGATACTACTACTTACA	Amp. HR2 1421800 Fw
9	1421800_+1555_Rv_InF	gaaaagtgccacctgacgtcAACATGTTTGCCTTAATACAATAAA	Amp. HR2 1421800 Rv
10	1421800_+23_sgRNA_Fw	taagtatataatattACATCCTTATAATAGTTACGgttttagagctagaa	sgRNA 1421800 Fw
11	1421800_+43_sgRNA_Rv	ttctagctctaaaacCGTAACTATTATAAGGATGTaatattatatactta	sgRNA 1421800 Rv
12	1421800_-588_Fw	CAAGTCTGTCCTACATACATAC	Ext. PCR 1421800 KO
13	1421800_+1783_Rv	CCCTATACAAATGTGTTTGAATT	Ext. PCR 1421800 KO
14	Seryl_+590_Fw	AAGTAGCAGGTCATCGTGGTT	qPCR seryl Fw
15	Seryl_+747_Rv	TTCGGCACATTCTTCCATAA	qPCR seryl Rv
16	Uce_+67_Fw	GGTGTTAGTGGCTCACCAATAGGA	qPCR uce Fw
17	Uce_+155_Rv	GTACCACCTTCCCATGGAGTA	qPCR uce Rv
18	hsp70-1_+1155_Fw	TGCAGCTGTACAAGCAGCC	qPCR hsp70-1 Fw
19	hsp70-1_+1315_Rv	GACTCTTTTTAGCAGGTATGG	qPCR hsp70-1 Rv
20	hsp86_+1860_Fw	ATCAGAATTTGGATGGTCCGC	qPCR hsp90 Fw
21	hsp86_+1989_Rv	TGATATAATTGGGTGACGAGC	qPCR hsp90 Rv
22	PF3D7_1421800_+217_Fw	GTTGATGATAATGTGAAAAACCG	qPCR 1421800 Fw
23	PF3D7_1421800_+350_Rv	TATAACGTGTGAAATTCAATATCC	qPCR 1421800 Fw
24	K13_+368_Fw	GCAAATCTTATAAATGATGATTCTGG	Amp. K13 locus Fw*
25	K13_+2471_Rv	GCTAATAAGTAATATCAATATAAGGG	Amp. K13 locus Rv*
26	K13_+1311_Fw	GGTATTAAATTTTTACCATTCCCATTAGTATTTTGTATAGG	Sanger seq. K13 mut Fw*
27	K13_+1937_Rv	TGTTCATTATCAATACCTCCAAC	Sanger seq. K13 mut Rv

*From Straimer et al. 2015, PMID 25502314

S1 Table. Primers used in this study.
